# Data Management in Health-Related Research Involving Indigenous Communities in the United States and Canada: A Scoping Review

**DOI:** 10.3389/fgene.2019.00942

**Published:** 2019-10-10

**Authors:** R. Brian Woodbury, Julie A. Beans, Vanessa Y. Hiratsuka, Wylie Burke

**Affiliations:** ^1^Research Department, Southcentral Foundation, Anchorage, Alaska, United States; ^2^Department of Bioethics and Humanities, University of Washington, Seattle, WA, United States

**Keywords:** indigenous people, American Indian, Alaska native, data management, data sharing, health research, community engagement, community-level research governance

## Abstract

**Background:** Multiple factors, including experiences with unethical research practices, have made some Indigenous groups in the United States and Canada reticent to participate in potentially beneficial health-related research. Yet, Indigenous peoples have also expressed a willingness to participate in research when certain conditions related to the components of data management—including data collection, analysis, security and storage, sharing, dissemination, and withdrawal—are met. A scoping review was conducted to better understand the terms of data management employed in health-related research involving Indigenous communities in the United States and Canada.

**Methods:** PubMed, Embase, PsychINFO, and Web of Science were searched using terms related to the populations and topics of interest. Results were screened and articles deemed eligible for inclusion were extracted for content on data management, community engagement, and community-level research governance.

**Results:** The search strategy returned 734 articles. 31 total articles were extracted, of which nine contained in-depth information on data management and underwent detailed extraction. All nine articles reported the development and implementation of data management tools, including research ethics codes, data-sharing agreements, and biobank access policies.

These articles reported that communities were involved in activities and decisions related to data collection (n=7), data analysis (n=5), data-sharing (n=9), dissemination (n=7), withdrawal (n=4), and development of data management tools (n=9). The articles also reported that communities had full or shared ownership of (n=5), control over (n=9), access to (n=1), and possession of data (n=5).

All nine articles discussed the role of community engagement in research and community-level research governance as means for aligning the terms of data management with the values, needs, and interests of communities.

**Conclusions:** There is need for more research and improved reporting on data management in health-related research involving Indigenous peoples in the United States and Canada. Findings from this review can provide guidance for the identification of data management terms and practices that may be acceptable to Indigenous communities considering participation in health-related research.

## Introduction

Indigenous groups located in the United States (U.S.) and Canada—including American Indian, Alaska Native, First Nations, Metis, and Inuit peoples—experience a disproportionate range of health disparities compared to other populations in these countries (McNally and Martin, 2017; IHS, 2018). Past experiences of harm and stigmatization resulting from unethical or misguided research practices have made some Indigenous communities mistrustful of research and consequently reticent to participate in health research despite the potential health benefits (Mello and Wolf, 2010; Pacheco et al., 2013). Although sharing of data for use in secondary research has been flagged as an area of particular concern for Indigenous people (James et al., 2014), individuals and communities have concerns about health research that extend beyond data-sharing to encompass data management as a whole (Hiratsuka et al., 2012b).

Data management refers to the policies, protocols, and practices related to data collection; analysis and interpretation; storage and security; sharing; withdrawal and disposal; and, return of results to participants and dissemination of results to the broader public (Michener, 2015; Pulsifer et al., 2011). These *components* of data management comprehend the range of data-related practices, protocols, and policies that can occur over the course of a research project. Data management is also inclusive of *principles* that delineate individual and community rights in relation to research data, including those that confer decision-making authority over access to and use of data. The data ownership, access, control, and possession (OCAP^TM^) principles developed by the National Steering Committee of the First Nations and Inuit Regional Longitudinal Health Survey recognize “First Nation jurisdiction over information about the First Nation” (First Nations Information Governance Centre, 2014, p. 36) and provide a valuable framework that can inform a rights-based approach to data management in research involving First Nations and other Indigenous groups (Schnarch, 2004).

Existing literature on the perspectives of Indigenous peoples in the U.S. and Canada indicates that the acceptability of research for these groups is contingent upon factors related to these components and principles of data management (James et al., 2014; Garrison et al., 2019). Issues of concern to Indigenous communities include: the types of data collected and how and from whom it is collected (Angal et al., 2016); the role of the community in the interpretation and analysis of data (Harding et al., 2012); the measures taken to maintain the security of data and the potential for loss of confidentiality (Sprague et al., 2013); the conditions under which biospecimens and other data types are stored (Hiratsuka et al., 2012a; Hiratsuka et al., 2012b; Haring et al., 2018); the terms of specimen withdrawal and disposal (Hiratsuka et al., 2012a; Hiratsuka et al., 2012b); with whom, for what purposes, and with whose permission data can be shared (Filippi et al., 2012); processes for returning results and progress reports to individuals participating in research (Hiratsuka et al., 2012a; Hiratsuka et al., 2012b); and, community involvement in the review and approval of dissemination products (Buchwald et al., 2006; Dirks et al., 2019).

Despite concerns related to data management, Indigenous groups in the U.S. and Canada have not enacted blanket proscriptions on research. Rather, communities have expressed a willingness to participate in research, provided certain conditions related to the components of data management are met (Jacobs et al., 2010; Bardill, 2017; Hiratsuka et al., 2017). Research approaches and regulatory tools that promote community engagement in and control over research processes—such as participatory research and community-level research governance—can aid efforts to shape data management terms in ways that meet these expectations (Manson et al., 2004; Harding et al., 2012; Mohammed et al., 2012).

Participatory approaches to research, including community-based participatory research and tribal participatory research, share a common emphasis on promoting community engagement in, control over, and benefit from research. Some Indigenous communities in the U.S. and Canada have indicated a preference for or explicitly recommended or required the use of such approaches in research (Burhansstipanov et al., 2002; Bharadwaj, 2014; James et al., 2014; Dillard et al., 2018). Community-level governance of research refers to the use of community-based mechanisms for guiding and regulating research. In the context of Indigenous communities in the U.S. and Canada, these can include tribal institutional review boards (IRBs), tribal advisory committees, research review committees, community advisory boards, formal tribal resolutions or agreements, and other regulatory and guidance groups, documents, and processes. As with community engagement, community-level research governance grants control over research processes, including data management terms and practices.

Crucially, these mechanisms have special legal and political force when they are implemented by Indigenous communities that are recognized as sovereign nations. In the U.S., there are 573 federally recognized tribes and over 200 tribes seeking federal recognition (Koenig and Stein, 2008; DHHS, 2018). In Canada, section 35 of the *Constitution Act, 1982* recognizes self-governance as an inherent right held by Indigenous groups (Government of Canada, 2019). Indigenous governments exercise their right to self-governance in the development and enforcement of mechanisms for guiding and regulating data management and other aspects of research (Hiratsuka et al., 2017; Garrison et al., 2019).

**Figure 1:**
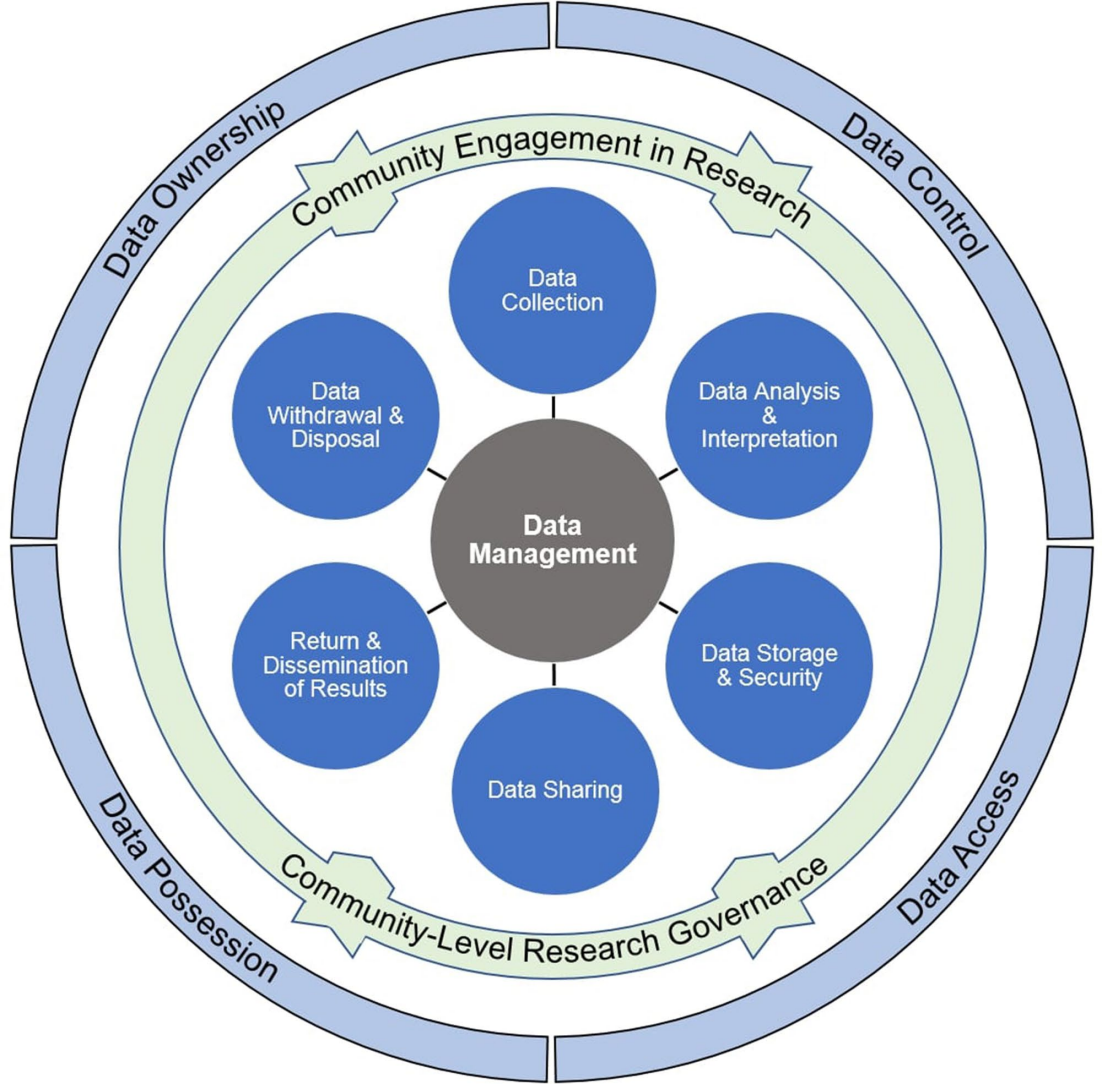
Data Management Conceptual Model. The model represents the relationship between data management principles (light blue outer ring), community engagement in research and community-level research governance (green inner ring), and data management components (dark blue circles). The six data management components comprise the full range of data management activities in research; this is represented by the spoke and hub relationship between individual data management components and the parent concept of data management. Arrows in the green ring indicate how data management principles are realized in data management practices, protocols, and policies by means of community engagement in research and community-level research governance.

The principles of data management, the components of data management, and community engagement in and governance of research are related but distinct elements of a conceptual model of data management (**Figure 1**). Data management principles articulate the rights of Indigenous communities to own and control their data and to decide the terms of data access and possession. These principles inform the range of data-related practices, protocols, and policies that are employed in a research project and that are associated with one or more data management components. Community engagement in research and community-level research governance are tools that communities can leverage to help ensure that the components of data management—as manifest in research practices, protocols, and policies—reflect the principles of data management.

Given the connections between the elements of data management depicted in **Figure 1** and the acceptability of research for Indigenous communities in the U.S. and Canada, detailed accounts of these elements as they occur in health-related research involving Indigenous communities have substantial practical value for multiple stakeholders and should be available through key sources of information, including the peer-reviewed literature. Yet, in-depth descriptions of data management in this area of research are uncommon in the peer-reviewed literature and reviews that synthesize this information do not exist. Templates and guidance documents for developing research codes and other research governance documents are available (American Indian Law Center, 1999), as are reviews of these guidelines and recommendations (Warren-Mears, n.d; Bardill, 2017), but these documents are either not peer-reviewed or are peer-reviewed but limited in their provision of information on the data management terms that have been used in health-related research involving the populations of interest. To address this research gap, we reviewed the scientific literature for articles providing detailed accounts of data management in health-related research involving Indigenous populations in the U.S. and Canada.

## Methods

Scoping reviews seek to summarize research findings; identify research gaps; determine the need for systematic reviews; and inform policy, practice, and research (Daudt et al., 2013). This scoping review employed the methodological framework initially developed by Arksey and O’Malley (2005) and subsequently refined by several research groups (Levac et al., 2010; Daudt et al., 2013; Peters et al., 2015).

### Research Question and Study Purpose

This review sought to answer the question: “How is data managed in the context of health-related research and related activities involving Indigenous populations located in the U.S. and Canada?”

For this study, “data” refers to any information or specimens collected for health-related research or related activities, including but not limited to demographic and genetic information and biospecimens. “Data management” is a categorical term inclusive of the data management components and principles described above. “Health-related research” refers to research efforts to assess or promote health and includes clinical, public health, and environmental health research as well as activities to develop public health surveillance infrastructure. Indigenous groups in the U.S. and Canada include American Indian, Alaska Native, First Nations, Metis, and Inuit peoples.

The purpose of this study is to survey the peer-reviewed literature on health-related research involving Indigenous populations in the U.S. and Canada in order to identify and report on detailed accounts of the conditions of data management in this research area, to identify opportunities and priorities for future research, and to offer recommendations for research practice.

### Research Team

The research team included three researchers in the Southcentral Foundation Research Department (RW, JB, VH) and one faculty member from the University of Washington’s Department of Bioethics and Humanities (WB). Southcentral Foundation is a tribally owned and operated health care organization headquartered in Anchorage, Alaska. VH, JB, and RW conduct team-based research on topics including the ethical, legal, and social implications of genomic research. VH and JB are Indigenous scholars. WB is a medical geneticist and bioethicist whose work involves study of the ethical and policy implications of genomic research.

### Search Strategy

PubMed, Embase, PsychINFO, and Web of Science were searched between July and September 2017 for articles describing data management terms and practices in the context of health-related research involving Indigenous communities in the U.S. or Canada. Results were not limited by date of publication. The search strategy included MeSH terms related to Indigenous populations in the U.S. and Canada and MeSH terms and keywords related to data management and associated topics. For each topical MeSH term and keyword, five test searches with increasingly restrictive criteria were run and the results scanned, with the goal of identifying the search that returned the largest number of results with face value relevance to data management. The best of the five test searches for each topical MeSH term and keyword was included in the final search strategy (**Supplement 1**), which included a total of 72 topical MeSH terms and keywords and 3 population MeSH terms. A medical librarian reviewed the final search strategy and did not suggest any modifications.

### Article Selection

**Figure 2:**
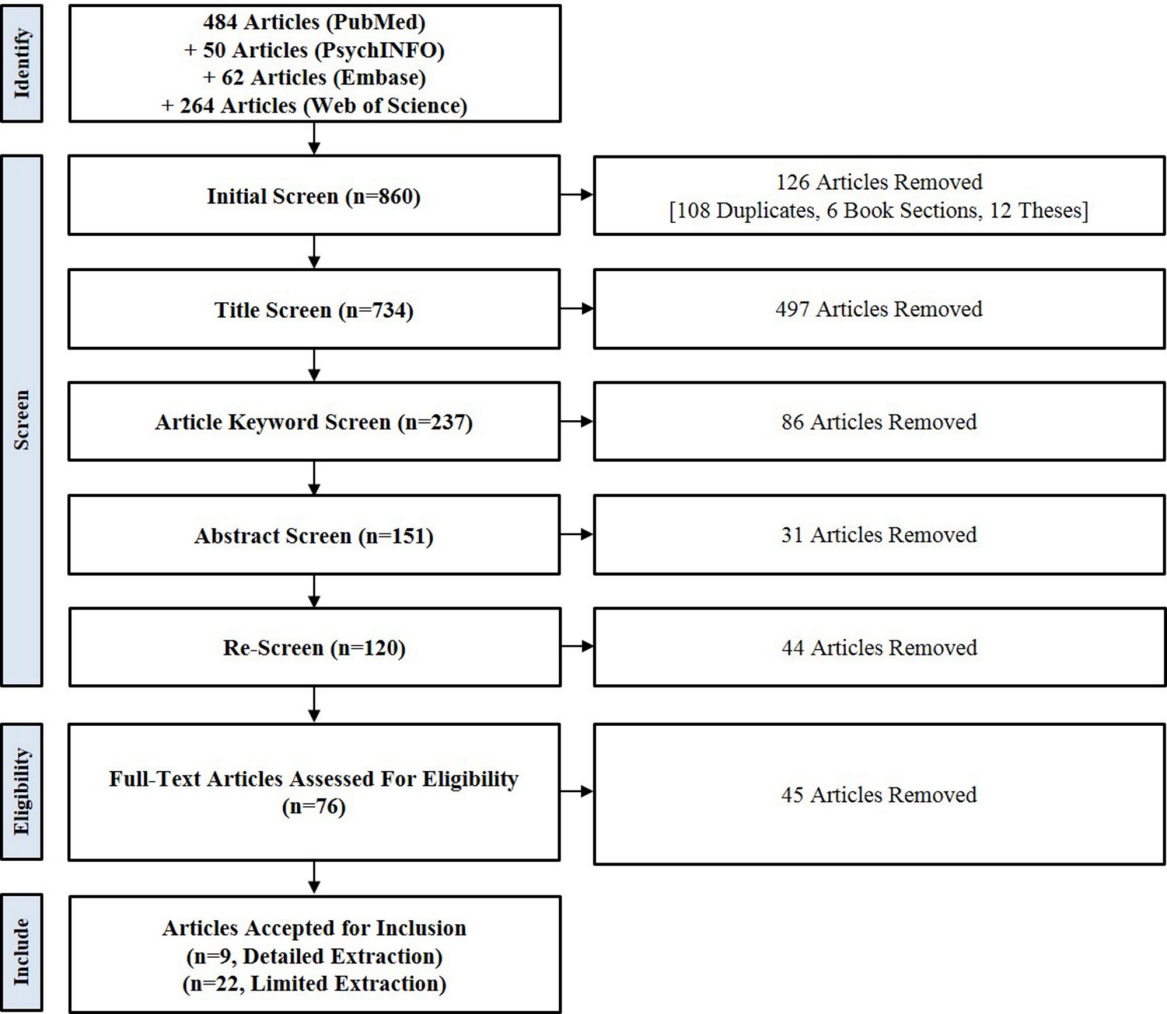
Scoping review search strategy.

We retained for extraction any English language, peer-reviewed, primary research article that included a detailed account of the terms of data management in health-related research involving AIAN communities. Secondary research articles (e.g., literature reviews, commentaries) were included if they contained in-depth discussion on issues related to or the conditions of data management in health-related research involving the populations of interest. Article selection was guided by a formal screening protocol (**Supplement 2**). Article titles, keyword lists, and abstracts were screened sequentially to determine relevance to the research question (**Figure 2**). RW and JB independently screened all articles and compared results. Articles deemed relevant by only one reviewer were rescreened by RW. The full text of the remaining articles were reviewed by RW and WB for the presence of information on data management terms and practices used in research involving Indigenous populations in the U.S. and Canada. Articles that included no information on these topics were removed. The remaining 31 articles were deemed eligible for inclusion in the review; of these, nine provided detailed information about the terms of data management and were selected for detailed extraction and 22 provided general information about data management, community engagement, or community-level research governance and were selected for limited extraction. The reference lists of the nine articles selected for detailed extraction were compiled and searched for additional relevant articles. No additional relevant articles were identified.

### Data Extraction

A detailed data extraction form was developed through an iterative process and with input from all authors. RW developed an initial draft, drawing on a preliminary review of the search results and lessons learned from an earlier scoping review (Woodbury et al., 2019). This draft was piloted using two articles from the search results and subsequently revised over the course of several team meetings. The final version of the detailed data extraction form (**Supplement 3**) includes 66 questions. Most questions include a set of standardized responses and inclusion/exclusion criteria or notes to guide the reviewer. The questions concern the elements of the data management conceptual model: the components and principles of data management, community-level research governance, and community engagement in research. The data extraction form also includes questions related to basic information about the articles (e.g., article purpose, study approach, and methods).

An abbreviated version of the data extraction form was developed to enable extraction of articles containing only general information or limited details about data management and related topics (**Supplement 4**). Based on the detailed data extraction from, this limited data extraction form includes 21 questions on data management, governance, community engagement, and basic information about the articles.

To assess the reliability of the extraction process, RW and JB extracted the same article using the detailed data extraction form and compared results; this process was duplicated by RW and WB for the abbreviated data extraction form. Results were comparable in both cases. Each of the remaining articles were extracted by either RW or WB.

## Results

After removing duplicates, the search strategy returned 734 unique articles: 484 articles from PubMed; 62 from Embase; 50 from PsychINFO; and, 264 from Web of Science. Seventy-six articles remained after the screening process, of which 31 were deemed eligible for inclusion in the review. Nine articles underwent detailed extraction, while 22 underwent limited extraction. We present an overview of the extraction results for all 31 articles included in the analysis, followed by results specific to the elements of the data management conceptual model that were assessed by the data extraction form. The sections below begin with a brief definitional statement of a specific element, followed by an overview of the treatment of that element across the reviewed articles, and then further detail and illustrative examples where available. **Table 1** provides an overview of results from all articles. **Tables 2** and **3** provide results only for those articles that underwent detailed extraction and focus on data management and on community engagement in data management and research regulation, respectively.

**Table 1:** Results Overview.

Author, Year	Article Type	Brief Description	Data Management, Data Management Tool	Research Governance, Research Governance Tool	Participatory Approach	Extraction
Scott and Receveur, 1995	C	Reviews ethical guidelines for research involving indigenous populations.	N, N	Y, N	N	L
Norton and Manson, 1996	C	Describes ethical issues in clinical research, including defining and engaging communities, community-level research review processes, culturally-specific understandings of confidentiality, and community benefit.	N, N	Y, N	N	L
Foster et al., 1998	E,C	Describes the content and development of a genetic research agreement.	Y, Y	Y, Y	Y	D
Macauley et al., 1998a	C	Describes the context for and development of a research code created for a diabetes prevention project.	Y, Y	Y, Y	Y	L
Macaulay et al., 1998b	C	Describes the development and use of a research ethics code.	Y, Y	Y, Y	Y	D
Lomawaima, 2000	C	Reviews long-term trends in research practices and power dynamics in relationships between researchers and AIAN communities.	Y, N	Y, N	N	L
Burhansstipanov et al., 2002	C	Describes genetic research issues and recommendations identified by American Indian tribal groups.	Y, N	Y, N	N	L
Sharp and Foster, 2002	R	Reviews research guidelines and presents recommendations for the ethical management of biospecimens.	Y, N	Y, N	N	L
Manson et al., 2004	R	Reviews examples of health-related research involving AI/AN communities that emphasized community engagement in and regulation of research.	N, N	Y, Y	Y	L
Burhansstipanov et al., 2005	C	Provides draft language for study protocols for genetic research.	Y, N	Y, N	N	L
Arbour and Cook, 2006	C	Describes ethical considerations in genetic research, including the distinction between data stewardship and ownership and the use of participatory approaches.	Y, N	Y, N	N	L
Brugge and Missaghian, 2006	C	Describes the Navajo Nation’s process for regulating research occurring within its jurisdiction.	N, N	Y, Y	N	L
Quigley, 2006	C	Reviews ethical practices in and provide guidelines and recommendations for environmental and public health research.	Y, N	Y, N	N	L
Boyer et al., 2007	C	Describes the use of a CBPR framework to guide dissemination of results findings from genetic research.	Y, Y	Y, Y	Y	D
DeLemos et al., 2007	E	Describes the role of community engagement in developing a culturally relevant approach to data collection in an environmental health study.	Y, N	N, N	Y	L
Minore et al., 2009	E,R	Describes a study to determine resources needed to develop a surveillance system for tracking the health status and health service utilization of indigenous populations in Ontario.	Y, Y	Y, N	N	L
Lauson et al., 2011	C	Describes the development of a maternal-child health surveillance system.	Y, Y	Y, Y	N	D
Pulsifer et al., 2011	C	Describes need for and potential challenges involved in the development of a database for indigenous knowledge.	Y, Y	Y, N	N	L
Flicker and Worthington, 2012	E	Describes perspectives of research ethics board members and other stakeholders on the review and conduct of public health research involving indigenous people.	Y, N	Y, Y	N	L
Harding et al., 2012	C	Reviews issues related to sovereignty, research ethics, and data-sharing and describes a material and data sharing agreement.	Y, Y	Y, Y	Y	D
Mohammed et al., 2012	C	Describes the development of a research protocol and data sharing agreement.	Y, Y	Y, Y	Y	D
Campbell, 2014	C	Describes issues related to data management and ownership in healthcare research involving indigenous people and research guidelines and principles developed to address these issues.	Y, N	Y, N	N	L
Geary et al., 2013	C	Describes issues related to data access and benefits sharing in research and provides recommendations for developing research agreements that address these issues.	Y, N	Y, N	N	L
Jiang et al., 2013	E	Describes the structure of a research-community partnership to implement a diabetes prevention program in AIAN communities and the outcome of this intervention.	N, N	Y, N	Y	L
Kelley et al., 2013	C	Describes the steps taken in assessing the need for and developing an inter-tribal IRB.	Y, N	Y, Y	N	L
Parkinson et al., 2013	C	Describes the development of biospecimen management policies for the Alaska Area Specimen Bank.	Y, Y	Y, Y	Y	D
Brunger and Russell, 2015	E,C	Describes how political conceptions and definitions of indigenous identity impact community representation in and authority over regulation of research involving indigenous people.	N, N	Y, Y	Y	L
Oetzel et al., 2015	E	Describes a study to characterize the relationship between community involvement in research regulation and community member and researcher perceptions of research processes and outcomes.	Y, N	Y, Y	N	L
Angal et al., 2016	C	Describes benefits of community participation in research ethics review processes.	Y, Y	Y, Y	Y	D
Brunger and Wall, 2016	E	Critiques approaches to community engagement in research that overburden and fail to empower communities and provides recommendations for engagement practices that contribute to ethical research.	Y, N	Y, Y	Y	L
Hardy et al., 2016	C	Describes a protocol to guide ethical data dissemination and other aspects of data management.	Y, Y	Y, Y	Y	D

C, Commentary; E, Empirical Study; R, Review; Y, Yes; N, No; D, Detailed Extraction; L, Limited Extraction; Data Management: Responds to the question “Is data management discussed in the article?”; Data Management Tool: Responds to the question “Does the article describe the use or development of any data management tools?”; Research Governance: Responds to the question “Is research governance discussed in the article?”; Research Governance Tool: Responds to the question: “Did the community participating in research employ its own methods for guiding/regulating research (e.g., tribal IRB, tribal resolution, CAB)?”; Participatory Approach: Responds to the question: “Were the research project(s) or program(s) described as employing a specific or a general participatory research approach?”; Extraction: Responds to the question “Did the article undergo limited or detailed extraction?”.

**Table 2:** Data Management.

Author, Year	Data Management Tool	Data Management Tool	Data Collection	Data Analysis and Interpretation	Data Security	Data Stored in Public Database	Data Sharing Occurred, Allowed	Return and Dissemination of Results	Data Withdrawal and Disposal	Total Data Management Components Reported per Article
Foster et al., 1998	Model Agreement for Genetic Research	collection storage/security dissemination sharing	biospecimen genetic other	NR	NR	NR	N, Y	Y	Y	4
Macaulay et al., 1998b	Code of Research Ethics	analysis dissemination	NR	Y	NR	NR	NR, Y	NR	NR	2
Boyer et al., 2007	Center for Alaska Native Health Research’s CBPR Approach to Genetic Research	collection analysis storage/security dissemination sharing	biospecimen genetic other	Y	NR	NR	N, Y	Y	NR	4
Harding et al., 2012	Material and Data-Sharing Agreement.	collection analysis storage/security withdrawal dissemination sharing	biospecimen other	Y	technical administrative physical	NR	N, Y	Y	Y	6
Mohammed et al., 2012	Research Protocol and Data-Sharing Agreement	collection analysis storage/security dissemination withdrawal sharing	other	Y	technical physical	NR	NR, Y	Y	Y	6
Lauson et al., 2011	Nutaqqavut (Our Children) Health Information System	collection sharing	other	NA	technical administrative	Y	NR, Y	NA	NR	3
Parkinson et al., 2013	Alaska Area Specimen Bank Policies and Procedures	collection storage/security withdrawal sharing	biospecimen genetic	NA	technical administrative physical	Y	NR, Y	NA	Y	4
Angal et al., 2016	Tribal Research Oversight Process	dissemination sharing	biospecimen genetic	NR	NR	NR	NR, Y	NR	NR	2
Hardy et al., 2016	Data Management Protocol and Permissions for Access and Use of Data Sets	analysis dissemination sharing	other	Y	other	N	NR, Y	Y	Y	6
Total Articles Reporting on Data Management Components	NA	NA	8	5	5	3	3, 9	5	5	

Y, Yes; N; NR, Not Reported; NA, Not Applicable; Data Management Tool: Responds to the question “If the article describes the use or development of any data management tools, indicate whether the tool involves or addresses data collection, data sharing, ownership/control of data, data storage/security, dissemination or return of results, and/or conditions for withdrawal of data.”*; Data Collection: Responds to the question “Did the research study or project/program collect biospecimens, genetic data, or data other than biospecimens, genetic data, or demographic data?”*; Data Analysis and Interpretation: Responds to the question “Does the article describe processes used to analyze or interpret data or results or the terms of data analysis/interpretation?”; Data Security: Responds to the question “Were technical, administrative, physical, or other security measures taken to secure participant data?” Data Stored in Public Database: Responds to the question “Is collected data stored in a public/private database/biobank?”; Data Sharing Occurred, Allowed: Responds to the questions “Did the article describe any data sharing activities?” And “Is sharing data for secondary research allowable?”; Return and Dissemination of Results: Responds to the question “Were study or project/program results/findings returned to participants or disseminated to the public?”; Data Withdrawal and Disposal: Responds to the question: “Did the article discuss processes related to the withdrawal of data from research or databases or the disposal or destruction of data?”; Total Data Management Components Reported per Article: Sums Y or equivalent responses from columns 4–10 (Note: Y responses in columns 6 and 7 are combined and count only once towards total; Y responses in column 8 are combined and count only once towards total). *These questions synthesize two or more discrete questions as presented in the data extraction form. See **Supplementary Materials** for details.

**Table 3:** Community Engagement.

Author, Year	Community Developed Data Management Tool	Community Engagement in Data Management	Community-level Research Governance	Community-level Data Ownership, Control, Access, and Possession
Foster et al., 1998	Y	storage sharing dissemination withdrawal	Y tribal IRB CAB other	control
Macaulay et al., 1998b	Y	analysis sharing dissemination	Y CAB	ownership control possession
Boyer et al., 2007	Y	collection analysis storage sharing dissemination	Y tribal resolution other	control
Harding et al., 2012	Y	collection storage sharing dissemination withdrawal	Y CAB other	ownership control possession
Mohammed et al., 2012	Y	collection analysis storage sharing dissemination withdrawal	Y tribal IRB tribal resolution CAB other	ownership control possession
Lauson et al., 2011	Y	collection sharing	Y	control access possession
Parkinson et al., 2013	Y	collection storage sharing withdrawal	Y CAB other	ownership control
Angal et al., 2016	Y	collection analysis sharing dissemination	Y tribal IRB tribal resolution CAB	ownership control possession
Hardy et al., 2016	Y	collection analysis storage sharing dissemination	Y CAB other	control

Y, Yes; N, No; NR, Not Reported; NA, Not Applicable; CAB, Community advisory board; Community-level Research Governance: Responds to the question “Did the community participating in research employ its own methods for guiding/regulating research (e.g., tribal IRB, tribal resolution, CAB)?”; Community Developed Data Management Tool: Responds to the question “If the article describes the use or development of any data management tools, was the community involved in the development or selection of the data management tools that were developed?”*; Community engagement in data management: Responds to the question “Was the community involved in activities or decisions related to data collection; data analysis and/or interpretation; data security and/or storage; data sharing for purposes of secondary research; dissemination of research results; or, withdrawal and/or disposal of data?” Community-level Data Ownership, Control, Access, and Possession: Responds to the question “Did the community retain ownership of, control of, access to, and/or possession of research data?”*; *These questions synthesize two or more discrete questions as presented in the data extraction form. See **Supplementary Materials** for details.

All reviewed articles: 1) described, evaluated, or reviewed health-related research or related activities involving Indigenous communities in the U.S. and Canada; and 2) included information about research activities and processes related to data management or about governance of research. The reviewed articles included 2 literature reviews, 5 empirical studies, 21 commentaries, and 3 articles with a hybrid design that combined empirical research with a commentary or literature review. Twenty-five articles discussed data management in general terms and 12 described the development or use of a data management tool (e.g., data-sharing agreement, databases/biobanks) for use in health-related research or related activities. Thirty articles included discussion on research governance; among these, 17 provided detailed descriptions of the development or use of a community-level mechanism for guiding or regulating research (e.g., tribal IRB, community advisory board). Fourteen articles reported the use of a participatory approach (e.g., community-based participatory research, participatory action research) to research.

All reviewed articles concerned genetic and public research broadly or reported on specific studies that addressed a range of health conditions and issues. Six articles described the development, purpose, and content of research codes, data sharing agreements, and other documents that set the terms of data management for a specific research project (Foster et al., 1998; Hardy et al., 2016; Macauley et al., 1998a; Macauley et al., 1998b; Harding et al., 2012; Mohammed et al., 2012). Seven articles reviewed existing research guidelines and provided template language for research protocols or recommendations for conducting ethical research and developing data management tools (Scott and Receveur, 1995; Burhansstipanov et al., 2002; Sharp and Foster, 2002; Burhansstipanov et al., 2005; Quigley, 2006; Geary et al., 2013; Campbell, 2014). Three provided detailed accounts of the development of tribal IRBs and other community-level groups and processes involved in research regulation (Brugge and Missaghian, 2006; Kelley et al., 2013; Angal et al., 2016). Two articles described the development of operational policies for databases or biobanks to support health research (Lauson et al., 2011; Parkinson et al., 2013) and two discussed challenges and considerations related to developing health information and Indigenous knowledge databases (Minore et al., 2009; Pulsifer et al., 2011). Three articles reported on participatory research approaches and community-researcher partnerships that considered components of data management (Boyer et al., 2007; DeLemos et al., 2007; Jiang et al., 2013). The eight remaining articles discussed considerations and stakeholder perspectives related to data management, research regulation, and community engagement (Norton and Manson, 1996; Lomawaima, 2000; Manson et al., 2004; Arbour and Cook, 2006; Flicker and Worthington, 2012; Brunger and Russell, 2015; Oetzel et al., 2015; Brunger and Wall, 2016). Articles described research involving Indigenous communities located in several U.S. states, including Alaska, Arizona, North Carolina, North and South Dakota, Oklahoma, Oregon; the Pacific Northwest, Southwest, and Great Plains regions of the U.S.; and, the Canadian provinces and territories of Labrador, Newfoundland, Nunavut, Ontario, and Quebec.

### Data Management Components

#### Data Collection

Data collection refers to the identification of relevant existing data and the creation and organization of new data and metadata. Articles that underwent detailed extraction were reviewed for content on the types of data collected (e.g., genetic data, biospecimens) and to determine whether any data management tools described in the article included details on data collection activities (**Table 2**). Five of these articles reported the collection of biospecimens (Harding et al., 2012; Foster et al., 1998; Boyer et al., 2007; Parkinson et al., 2013; Angal et al., 2016) and four reported the collection of genetic information (Foster et al., 1998; Boyer et al., 2007; Parkinson et al., 2013; Angal et al., 2016). Six articles reported the collection of other types of data. For example, data on maternal environmental exposures, diet, health behaviors, social determinants, and health status during prenatal, perinatal, and postnatal periods was collected for a maternal-child health information system (Lauson et al., 2011). Articles also reported the collection of diet, exercise, and psychosocial data (Boyer et al., 2007); transcripts and other qualitative data from interviews, focus groups, and a public seminar (Hardy et al., 2016); and, survey data (Mohammed et al., 2012).

Articles that underwent limited extraction described how cultural beliefs influence the types of data that are appropriate for use in research. For example, Arbour and Cook (2006) and Burhansstipanov et al. (2005) note that blood and hair are sacred to some Indigenous communities and may require special handling during collection and that researchers unaware of these beliefs have caused offense by requesting the use of hair samples for research. In other communities, beliefs requiring that the body remain whole after death preclude the collection of biospecimens from deceased persons (Burhansstipanov et al., 2005). Cultural beliefs also affect the feasibility and acceptability of data collection methods. For example, Foster et al. (1998) report that, due to cultural conventions blood samples were collected by an individual from outside the community. Sharp and Foster (2002) recommend taking account of factors such as religious holidays and expectations about privacy when determining the timing and location of data collection efforts.

Data collection is also affected by culturally-informed conceptions of knowledge. Campbell (2014) observes that sources of legitimate knowledge for Indigenous cultures can include “place, cosmology, elders, dreams, visions, and paths” (p. 41). Researchers may not recognize the epistemic value of these knowledge sources, with the result that information deemed relevant by the community may not be captured by researchers or handled in accordance with data collection protocols. Differences between some types of traditional knowledge and the types of data commonly used in health research can also create technical issues when translating between these types of information. For example, Geary et al. (2013) note that there are difficulties involved in reducing traditional knowledge into data points in a database, due to its dynamic, varied, and contextual nature.

#### Data Analysis and Interpretation

Data analysis and interpretation refers to the process of synthesizing, manipulating, and interpreting data to arrive at results. Articles that underwent detailed extraction were reviewed for content on analysis and interpretation of data and results and to determine whether the community was involved in these activities and whether any data management tools described in the article included details on data analysis and interpretation (**Table 2**). This content was not present in the articles that underwent limited extraction.

Information on the analysis and interpretation of data and results was somewhat limited. Among articles that underwent detailed extraction, four articles—including two that concerned databases or biobanks rather than research—did not describe the terms of data analysis or interpretation and five did not report whether the community was involved in these components of data management.

Boyer et al. (2007) state that interpretation of results was collaborative in nature and involved community members and staff of a tribally-owned regional health corporation. Mohammed et al. (2012) report that community members and researchers were involved in the co-analysis of data for a project to prevent cardiovascular disease. In a study on health resilience, community members were hired onto the research team, trained in data analysis methods, and participated in the selection, analysis, and reporting of salient themes from the qualitative data (Hardy et al., 2016). Macauley et al. (1998b) state that involving community members in interpretation of results can contribute to “richer contextual information and increasingly meaningful conclusions” (p. 105) and describe a local community advisory board as taking an active role in this component of data management.

In addition to these project-specific accounts of community involvement in data analysis, three articles noted that participatory approaches to research call for community involvement in data analysis and interpretation (Macauley et al., 1998b; Angal et al., 2016; Hardy et al., 2016) one suggests that community members be involved in the interpretation of preliminary research activities to evaluate community decision-making structures (Foster et al., 1998); and Harding et al. (2012) remark broadly that the “conditions for data analyses, including scope of research, privacy issues, and [intellectual property rights]” (p. 7) should be mutually agreed upon by researchers and the community.

In some cases, the terms of data analysis and interpretation were made explicit by the language of data management tools. For example, the research agreement described by Mohammed et al. (2012) lists the requirements for conducting any subsequent analyses with existing data. The code of research ethics referenced in Macauley et al. (1998b) describes the community’s role in interpreting the results as an obligation and states that academic researchers, community-based researchers, and community members retain the right to disagree with the interpretation of results presented in a manuscript or other research product and to submit a dissenting opinion. Harding et al. (2012) report on a material and data-sharing agreement that contains details on all planned analyses, including the purpose of and datasets involved in each analysis.

Although the need for data management and/or research governance tools to control secondary use of data was a common theme across articles, Hardy et al. (2016) were unique in describing the risks associated with secondary use in terms of potentially stigmatizing reanalysis and reinterpretation of data and in developing a data management protocol specifically to protect against these risks. They observe that their ability to build a trust-based relationship with the community was contingent upon the use of a strengths-based rather than a deficits-based framework to guide their research, with the implication that the lens through which data were analyzed and interpreted mattered as much as the research question itself. Permitting other investigators to use data for secondary research without regard to the nature of the analysis framework to be utilized would violate community trust and could lead to misinterpretation of data and community stigma.

#### Data Storage and Security

Data storage refers to the act of organizing and maintaining records with the goal of preserving the retrievability and integrity of data for archival and other purposes. Data security refers to the use of technical, administrative, and physical controls to protect data from unauthorized access, use, and alteration. Technical controls involve the use of technology to control access to and use of data and include computer passwords and file encryption. Administrative controls are the policies and protocols impacting human behavior as it relates to data security and include rules that restrict access to data to specific individuals. Physical controls are methods for controlling physical access to data and include storing data in restricted access facilities or in locked filing cabinets. The U.S. Department of Health and Human Services uses this tripartite taxonomy in its description of the data security requirements under the Health Insurance Portability and Accountability Act’s Security Rule (DHHS, 2013).

Articles that underwent detailed extraction were reviewed for content on the methods used to de-identify data, the storage of data in databases or biorepositories, and the use of technical, administrative, and physical security controls taken to secure research data. Four articles described the use of technical controls, including encrypted and pass-word-protected digital media and a restricted access website to facilitate data-sharing across research sites (**Table 2**). Administrative and physical controls were described in three articles each and included the use of locked filing cabinets and offices and laboratories accessible only by coded entry and restricting access to data to those members of the research team who signed a confidentiality agreement. Only two articles described the use of all three categories of security controls.

Six articles provide insufficient information to determine whether data was stored in a publicly accessible biobank or other data repository; one article explicitly stated that data was not housed in this manner (**Table 2**). In their descriptions of a specimen bank and heath surveillance database, Parkinson et al. (2013) and Lauson et al. (2011) provide details relevant to data storage and security. Specimens stored in the Alaska Area Specimen Bank are kept in lockable freezers located in the controlled-access laboratory at the Centers for Disease Control and Prevention’s Arctic Investigations Program facility (Parkinson et al., 2013, S3). Specimen labels do not include any information beyond a numerical identifier, specimen type, and date of collection (Parkinson et al., 2013, S14). In their description of the development of the Nutaqqavut Health Information System, Lauson et al. (2011) state that data would be de-identified, that no individual-level data would be accessible from the database, and that this system was approved by provincial government authorities under relevant privacy laws.

Six articles explicitly stated that data would be deidentified at the individual-level (Foster et al., 1998; Boyer et al., 2007; Harding et al., 2012; Lauson et al., 2011; Mohammed et al., 2012; Parkinson et al., 2013); the remaining three articles did not report this information (Macauley et al., 1998b; Angal et al., 2016; Hardy et al., 2016). Deidentification at the community-level was also a concern. For example, Boyer et al. (2007) explain that the communities participating in their research preferred to be identified by region only, in order to better protect the identity of individual villages. In addition, Foster et al. (1998) state that the community was empowered through its review process to anonymize research products at the community-level. Three articles described participating communities in terms of the regions (e.g., Southwest, Pacific Northwest) in which they were located (Boyer et al., 2007; Hardy et al., 2016; Mohammed et al., 2012), but provided no further identifying information, four articles identified the specific communities involved in research (Foster et al., 1998; Macauley et al., 1998b; Harding et al., 2012; Angal et al., 2016), and two articles did not concern research with specific communities (Lauson et al., 2011; Parkinson et al., 2013).

Among articles that underwent limited extraction, Burhansstipanov et al. (2005) provide draft policy language requiring that specimens collected for research be stored for the duration of the study only and then withdrawn and returned to the tribe or disposed of in an appropriate manner. While also requiring that samples be deidentified—to include removal of individual, tribal, and geographic identifiers—they recognize how this constrains the ability of researchers to return samples at the request of participants.

#### Data Sharing

Data sharing refers to actions to make data and results accessible to other investigators for secondary research. Articles that underwent detailed extraction were reviewed for content on data sharing, with a focus on the permissibility of and control over data-sharing in support of secondary research.

Three of the nine articles that underwent detailed extraction reported that sharing of data for use in secondary research did not occur and six articles provided insufficient detail to determine whether these activities took place (**Table 2**). However, data-sharing was allowable under the data management tools described in all nine articles, although the circumstances under which sharing could occur varied. Five articles stated that data sharing for secondary research required the approval of both researchers and community representatives or participants (Macaulay et al., 1998b; Boyer et al., 2007; Mohammed et al., 2012; Angal et al., 2016; Hardy et al., 2016) and three articles stated that only participants or individuals or groups representing the participating community were involved in the approval process (Foster et al., 1998; Harding et al., 2012; Parkinson et al., 2013).

The two articles concerned with the policies of health information databases and biobanks provided the most detailed accounts of data-sharing terms. Sharing of biospecimens held in the Alaska Area Specimen Bank requires review and approval of research proposals by the specimen bank committee and the tribal health organization(s) representing the individuals whose samples are requested (Parkinson et al., 2013). In addition, consent for proposed research is required from the individual, except where the research is limited to anonymous testing. Lauson et al. (2011) stated that data held in the Nutaqqavut Health Information System would be shared with community members, research, and health care providers to answer research questions approved by a research advisory board and with the permission of the Nunavut Research Institute, a research licensing organization. In determining whether to approve a research question, the research advisory board would consider the “interests of the residents of Nunavut” (p. 369), but the membership of the board—and whether it includes community members or researchers—is not disclosed.

Among articles that underwent limited extraction, Burhansstipanov et al. (2005) and Sharp and Foster (2002) emphasized the connection between data-sharing and informed consent. In the former article, Burhansstipanov et al. (2005) report that among the tribes encountered in their research there is near consensus that the use of specimens must be restricted to the study for which they were collected and that specimens can only be accessed by individuals listed on the consent form and cannot be transferred to other individuals or groups without prior approval of the appropriate tribal research governance bodies. In cases where permission to use specimens for secondary research is sought, reconsent must be active—that is, participants must explicitly agree to the research and cannot be “opted-in” to secondary research by the language of the consent form. By contrast, Sharp and Foster (2002) acknowledge that active reconsent places burdens on participants that could be alleviated through use of a community-based representative responsible for determined the appropriateness of the use of data for a given secondary research purpose.

#### Return and Dissemination of Results

In this context, return of results refers to the process of making research results available to individuals and communities participating in research, while dissemination of results refers to the act of making general research findings available to the broader public. Articles that underwent detailed extraction were reviewed for content on return of results to individual and communities participating in research and community involvement in and control over the review and approval of research publications and presentations intended for the broader public.

Five of nine articles reported that research findings were returned to individuals and/or communities participating in research or disseminated to the broader public. Two articles did not report information on return or dissemination of results and questions on return of results were outside the scope of the two articles addressing development and/or operational policies of databases or biobanks, though it is noteworthy that neither article described policies relevant to returning results or discussed the issue in any way.

Six articles reported that communities were involved in the review and approval of research products intended for dissemination to the broader public and one article did not report this information. In addition, two articles reported that communities had control over decisions to publicly release research products, one article stated that the community did not have unilateral control over dissemination, and four articles did not report this information. The issue of results dissemination was outside the scope of the two articles concerning databases or biobanks.

The role of community members and other research stakeholders in the review and approval of research products for public dissemination varied. The Material and Data Sharing Agreement described by Harding et al. (2012) requires the review and approval by a tribal representative of all “publications and presentations developed using materials or data collected under this agreement” (p. S4) prior to dissemination. By contrast, Foster et al. (1998) describe a community review process narrowly focused on the acceptability of identifying the participating community in manuscripts. Researchers were required to respond to community objections specific to this issue, but it is not clear if the scope of the review process or of the committee review board’s authority extended to other aspects of a research product. In another case, a research review committee comprised of both academic researchers and community representatives was responsible for the review and approval of dissemination products and for ensuring that results are provided to the community (Mohammed et al., 2012). One article described a community-level review process where community approval was not required. Macaulay et al., 1998b state that a code of research ethics developed by community members and researchers provides all research partners (i.e., community representatives, community-based researchers, academic researchers) with the right to review—but not to approve—research products. Instead, the code describes a “right to dissent” that provides any research partner who disagrees with the content of a research product to publish a dissenting opinion concurrently with that product. Finally, some articles simply did not report whether community approval was required. In their description of community-level ethics review in the multi-site Safe Passage Study, Angal et al. (2016) state that the Oglala Sioux Tribe Research Review Board requested that researchers submit a lay summary of manuscripts intended for publication and remain available to respond to questions from Board members. However, the authors do not report whether manuscripts required Board approval in order to be submitted for publication.

#### Data Withdrawal and Disposal

Data withdrawal involves the removal of data from study records, often at the request of research participants. The related concept of data disposal involves the destruction of data and is sometimes used when data withdrawal is not feasible. Articles that underwent detailed extraction were reviewed for content on withdrawal of data from research, processes for disposal of data, and the role of the community in activities and decisions related to data withdrawal and disposal. Five of these articles discussed data withdrawal and disposal and four described how communities or individual research participants were involved in these activities.

Foster et al. (1998) describe how they offered participants the opportunity to withdraw their samples from research within 60 days of collection in order to protect individual autonomy against social pressures to participate in research. The research protocol and data sharing agreement detailed by Mohammed et al. (2012) included “regulations regarding the appropriate handling and destruction of biological samples” (p. 121) and timelines for data destruction.

Parkinson et al. (2013) provide extensive detail on the policies and procedures for the withdrawal and disposal of specimens stored in the Alaska Area Specimen Bank. For primary research involving collection of biospecimens, any specimens remaining upon study completion are discarded unless informed consent documents specifically allow for long-term storage. For secondary research involving the use of previously banked biospecimens, any leftover specimens are returned to the Alaska Area Specimen Bank or reported as destroyed by the principal investigator. Additionally, individuals reserve the right to have their banked specimens removed from storage and destroyed at any time.

Although Macauley et al. (1998b) and Angal et al. (2016) did not discuss procedures for data withdrawal or disposal at the request of individual research participants, they made clear that data would be returned to the community after study completion. Similarly, Harding et al. (2012) state that all data collected for research would be returned to the tribe upon study completion or termination and that the research institution would confirm in writing the destruction of all data copies. Finally, Hardy et al. (2016) note that disposal of data has both costs and benefits, in that disposal limits the value of data for researchers by preventing its use in subsequent research, but also protects against potential use of data in a manner that exposures participants to privacy risks and harm.

### Data Management Principles

Data can be owned by an individual (e.g., a research participant from whom the data was collected) or by a group (e.g., a community participating in research). Data control refers to the power to determine the fate of data, including whether and how it is collected, used, and shared. Data access refers to the ability of individuals and groups to view their data. Data possession refers to the physical control over data; it is the concrete realization of data ownership. The concept of possession recognizes that data may be held in trust by a data steward while remaining the property of the data owner. Originally described by the National Steering Committee of the First Nations and Inuit Regional Longitudinal Health Survey as a set of principles that affirm the right of their communities to own, control, access, and possess data derived from their cultural knowledge (First Nations Information Governance Centre, 2014), these principles also have relevance for other Indigenous groups.

All nine articles that underwent detailed extraction included some discussion of data ownership, control, access, or possession, but only three articles discussed all four principles. The terms of data control were discussed in 9 articles, while data ownership and possession were discussed in 7 articles each. Data access was discussed in five articles. These discussions did not always clearly specify whether communities retained ownership of, possession over, access to, and possession of data collected for research (**Table 3**). Nine articles reported that communities retained unilateral or shared control over data. Five articles each reported that communities or individuals participating in research retained ownership and possession of their data. Information confirming whether communities or individuals retained access to research data was particularly scarce, with only one article unequivocally reporting such retention.

Across these articles, the terms of data ownership, control, access, and possession were varied and nuanced, as were the relationships among these concepts and the rights and responsibilities they were seen to confer. For example, Mohammed et al. (2012) report that, while community representatives and researchers agreed that the community retained ownership of data collected from its members, the two groups initially interpreted “ownership” in different ways. The community representatives assumed that ownership entailed storage of data in community facilities and community control over access to data. By contrast, researchers assumed that data would be housed in secure data storage facilities at their academic institution in order to minimize privacy risks and to better enable analysis. After clarifying discussions, it was jointly decided to house data within university facilities until it could be stripped of identifying information. Deidentified data would then be duplicated and stored in community and academic facilities, with access controlled by a research review committee comprised of three community members and two researchers. The committee also reviewed and approved manuscripts and other public dissemination products.

Macaulay et al. 1998b describe similar circumstances wherein community retained ownership of data, but researchers—who were charged with maintaining data security—were in possession of the data throughout the study. In this instance, a duplicate dataset was not created; instead, the data was returned to the community upon study completion. Control of data was also nuanced, with the community maintaining unilateral control over some but not all aspects of data management. The community acted alone in deciding on the use of data for subsequent research, but control over the addition of staff members with access to data to the original research team was shared among academic researchers, community researchers, and the community. In addition, the code of research ethics developed for this study ensures that community members are involved in the review of all research products (e.g., scientific publications) and provided with an opportunity to publish a dissenting opinion. However, the code does not give the community the power to veto publication. In both Mohammed et al. (2012) and Macauley et al. 1998b, although the community was recognized as the sole owner of research data, community members and researchers shared decision-making power over data use and dissemination of results.

The policies guiding management of biospecimens held by the Alaska Area Specimen Bank state that specimens remain the property of the individual from whom they were collected and describe the Alaska Area Specimen Bank itself as “the property of the Alaska Native people [to] be used to benefit the health and well-being of Alaska Native people” (Parkinson et al., 2013, p. S5). For this reason, individuals control the use of their samples for research purposes and retain the right to have their samples withdrawn from the specimen bank (Parkinson et al., 2013, p. S5, p. S7-9). However, individuals may not access their banked specimens and biohazard regulations do not permit the return of specimens to individuals. In this case, recognition of ownership of specimens by the individuals from whom they were collected confers control over—but not access to or possession of—their data. By contrast, Angal et al. (2016) note that, as an expression of the rights of ownership, tribal communities participating in the PASS study require both the eventual return of research data to the community and tribal approval for any subsequent use of data. Here, community ownership of data ensures control over use of data in research and possession of data upon study completion. The status of community access to data during the course of this study was not mentioned.

The Material and Data Sharing Agreement described by Harding et al. (2012) provides the clearest and most complete account of the terms of data ownership, control, access, and possession. This document states that data and material provided to or collected by researchers is property of the tribes participating in research; that data sharing for purposes of secondary research requires written permission of the tribes and the dissemination products must receive approval from a tribal representative prior to publication; that access to data is limited to members of the research teams who have signed confidentiality agreements and submitted those agreements to the tribes; and, that all data must be returned to the tribe upon study completion.

This robust articulation of community rights over research data contrasted with the more limited conception of community ownership, control, access, and possession of data described in other articles. For example, Lauson et al. (2011) do not discuss ownership of data held in the Nutaqqavut Health Information System and community and individual control over this database is indirect and limited to community representation on a research advisory board and a subcommittee charged with oversight of the Nutaqquvut Health Information System activities. Additionally, individual access to data held in the system is not permissible, although the authors state that aggregate-level data is available to communities and other stakeholders for purposes of promoting maternal–child health. Foster et al. (1998) extend discussion of ownership to intellectual property derived from biospecimens collected for research. In their study, the sponsoring institution would retain primary ownership of all intellectual property produced from study data, but the participating community would receive 30% of profits resulting from the sale of this property.

Articles also described the role of data management protocols and participatory research approaches in promoting community ownership, control, access, and possession of data. For example, Hardy et al. (2016) note that data management protocols can provide protections for participants and their communities by anticipating and avoiding questions and conflicts related to data ownership. Boyer et al. (2007) describe the use of a participatory research approach to “reach a consensus on how to share power and control over specimens and information” (p. 23). As result of this approach, communities participated in the development of rules guiding use and storage of data and all research proposals and products required the approval of the local tribal health organization’s research review committee. For new research proposals unrelated to the health conditions addressed by the original research project, the consent of individuals whose samples were to be used was also required.

### Community Engagement in Research and Community-Level Research Governance

Articles that underwent detailed extraction were reviewed for content on research approach and community engagement in activities related to data management and on the development or implementation of community-level mechanisms for guiding or regulating research.

Eight out of nine articles that underwent detailed extraction reported the use of a participatory research approach (**Table 1**). Of these, five used a specific participatory research approach—including community-based participatory research (Boyer et al., 2007; Harding et al., 2012; Mohammed et al., 2012; Hardy et al., 2016) and community-based research (Angal et al., 2016)—and three used general participatory research approaches that promote community engagement and representation in research processes (Parkinson et al., 2013), that utilize communal discourse to engage community members (Foster et al., 1998), or that emphasize collaboration, co-learning, and community benefit (Macauley et al., 1998b).

Community engagement varied across the components of data management. All nine articles reported community involvement in the development of data management tools, including research codes and agreements; data sharing agreements; data management protocols; health surveillance databases; and, biobank specimen access protocols (**Table 3**). Articles reported community involvement in activities or decisions related to data management, though such involvement varied across components (**Table 3**). Seven articles reported community involvement in activities or decisions related to data collection, seven reported community involvement in the review and approval of research results and products for public dissemination, five reported community involvement in the analysis or interpretation of data or results, and respectively six and three articles reported community involvement in storing or securing data and in withdrawal or disposal of data. Community involvement was most common for activities and decisions related to data sharing (reported in nine articles).

Community engagement in data management took many forms, but most often involved participation in the development of data management tools. For example, representatives from tribal health organizations were among the members of the Alaska Area Specimen Bank Working Group tasked with developing the policies and procedures to guide management of banked specimens (Parkinson et al., 2013) and the material and data-sharing agreement described by Harding et al. (2012) was developed with input from tribal researchers, the tribal health commission, and the tribal advisory committee. Individual community members also shaped the terms of data management through direct participation in research activities. For example, Hardy et al. (2016) report that community members were trained in research methods and actively contributed to the collection and analysis of qualitative data. Boyer et al. (2007) describe a process for disseminating research results back to the community that involved the successive review and revision of presentation materials by community research assistants and community member focus groups.

Communities employed several methods for guiding and regulating the management of research data (**Table 3**). All nine articles that underwent detailed extraction reported the use of at least one community-level mechanism for guiding or regulating research and seven articles reported the use of two or more mechanisms. Seven articles reported the use of community advisory boards (CABs), three reported tribal institutional review boards (IRBs), and three reported that tribal approval or a formal tribal resolution was provided for the study. Other community groups responsible for guiding and regulating research included research advisory committees (Lauson et al., 2011), working groups (Parkinson et al., 2013), tribal research review boards (Angal et al., 2016), and tribal advisory committees and health commissions (Harding et al., 2012) among others. Additionally, the interrelatedness of data management and research governance meant that some data management tools also contributed to broader community efforts to guide and regulate research. For example, the data-sharing agreements described by Mohammed et al. (2012) and Harding et al. (2012) delineate the roles and responsibilities of community members and groups involved in the review of dissemination products.

These community groups were involved in a range of research oversight activities relevant to data management. Such activities frequently included reviewing and approving manuscripts and other research products for public dissemination (Boyer et al., 2007; Mohammed et al., 2012), approving requests to share data for secondary research (Lauson et al., 2011; Macauley et al., 1998b), and representing community interests in the development of data management tools (Harding et al., 2012; Parkinson et al., 2013). Tribal health organizations and CABs were sometimes involved in interpreting research results (Macauley et al., 1998b; Boyer et al., 2007), tribal committee review boards participated in negotiations over long-term storage of biospecimens (Foster et al., 1998), and Angal et al. (2016) describe how a tribal review board helped develop consent forms that provided participants with the options to permit or refuse the collection of specific types of data.

## Discussion

### Summary and Interpretation of Findings

Although the scientific literature has often emphasized the importance of accounting for community perspectives as they relate to sharing research data and disseminating research results, the Indigenous communities described in these articles were interested in participating in the development of policies and protocols guiding all components of data management. These components, although conceptually discrete, are interdependent in practice. Thus, the acceptability of sharing research data, like the acceptability of the larger research projects in the context of which data sharing takes place, is partly contingent upon other aspects of data management. Our findings suggest that the acceptability of the research projects described in the reviewed articles for the communities considering participation in those projects hinged in part on whether proposed data collection methods accounted for the cultural or spiritual significance granted to specific types of biospecimens by some communities; whether community perspectives informed the analysis and interpretation of data; whether security measures and conditions of storage provided adequate protections against privacy loss and consequent harm; whether community control over data extended to review of new research proposals and dissemination of research results; whether the terms of data withdrawal and disposal fully accounted for the interests of research participants and community-specific cultural beliefs; and, the extent of community engagement in decisions and activities related to each of these data management components.

Descriptions of the terms of data ownership, control, access, and possession were nuanced and sometimes ambiguous and incomplete. Community and individual ownership of data was explicitly recognized in several articles and seemed to confer some control over the uses and fate of data, but not necessarily possession of or access to data. Community control over data was rarely described as comprehensive or unilateral; instead, control was most often in relation to specific activities (e.g., data sharing and results dissemination) and shared among stakeholders with potentially dissimilar or even competing interests. The conditions, if any, under which individuals and communities participating in research could access their data were underreported. Possession of data often transferred from communities to research institutions, who functioned as data stewards for the duration of the study and were expected to return data to communities upon study completion. The account provided by Mohammed et al. (2012) of identifying and overcoming differences in how researchers and community members understood data ownership and it implications for data control, access, and possession provides an object lesson in the complexity and interrelatedness of these concepts and an example for how to arrive at mutually acceptable terms of data management.

Our findings support the relationships between community engagement in research, community-level research governance, and the components and principles of data management that are depicted in the conceptual model of data management informing this review. In particular, community engagement in research processes—especially in activities and decisions related to the components of data management—appears to lend itself to the realization of terms of data management that clearly affirm community rights over research data and community-level mechanisms for guiding and regulating research provide the formal means by which such terms are executed and enforced. In this way, community engagement in and governance of research can plausibly be construed as preconditions for the development of data management practices and policies that respect the right of Indigenous communities to set the terms of data ownership, control, access, and possession in research.

There is a sizeable literature describing the benefits to communities of governing and engaging in research. In particular, the use of participatory research approaches as a means to promote community engagement has been widely recommended in health-related research involving Indigenous populations (Beans et al., 2019). Similarly, a substantive and growing literature describes the rights of self-governance and self-determination held by Indigenous populations in the U.S. and Canada and the legal and political force these rights give tribal IRBs and other individuals and groups empowered by tribal governments to regulate research (Hiratsuka et al., 2017). By contrast, the role of data management in shaping research practices in ways that benefit and empower communities has received comparatively little attention and warrants further investigation.

There was some evidence of a trend towards increased community oversight of and control over health-related research involving Indigenous peoples in the U.S. and Canada. This shift is reflected in the broader health research literature (Manson et al., 2004; Chino and Debruyn, 2006; Hiratsuka et al., 2017; Garrison et al., 2019). As highlighted in the results section, Macauley et al. (1998b) describes a research code that may limit the ability of the Indigenous community to oversee the dissemination of research results and Foster et al. (1998) describe how then current practices presumed ownership by the institution sponsoring research of intellectual property derived from study data. By contrast, Harding et al. (2012) clearly affirm community ownership of and control over research data and Boyer et al. (2007), Angal et al. (2016), and Hardy et al. (2016) describe research approaches and mechanisms of research governance that go further towards supporting the right of Indigenous communities to regulate research in which they participate.

The reviewed articles reported a wide range of community questions and concerns related to the components of data management (**Box 1**). Conducting research in a way that benefits and empowers communities and that accounts for community perspectives on data management requires collaboration among researchers and communities to answer and address these questions and concerns.

Box 1 **Community Questions About Data Management in Health-Related Research**

- What types of data are collected from whom using what methods?
- How are data analyzed and interpreted and what stakeholders are involved in these activities?
- Where and under what conditions are data stored?
- What technical, physical, and administrative controls are employed to secure data?
- With whom, for what purposes, and with what stipulations can data be shared?
- What is the process for returning results to individuals and communities participating in research and for disseminating results to the broader public?
- How are data withdrawn from research and how are they destroyed or otherwise disposed of?
- What are the terms of data ownership, control, access and possession?
- What is the nature of community engagement in activities and decisions related to data management?
- What community-level mechanisms for guiding and regulating research are employed?
- How are the terms of data management reported in the scientific literature and to communities?

### Areas of Opportunity for Future Research

One goal of a scoping review is to identify gaps and corresponding opportunities in research practice and policy. We identified three opportunities for future research and improving research practice and policy.

First, there is need for more complete descriptions of the data management terms and practices actually employed in research involving Indigenous communities in the U.S. and Canada. Despite searching four databases, we found only nine topically relevant articles that provided detailed accounts of the terms of data management. Even within this set of articles selected for their emphasis on data management, descriptions of activities and guidance documents related to data management practices were often sparse. The articles did not provide the information necessary to respond to 25 out of 72 questions on components of data management listed in **Table 1**. Given the role of data management in maximizing the benefits and minimizing the risks of research for communities and the potential of data management practices to alternately support or undermine community control over, access to, and ownership and possession of data, there is need for detailed reporting on data management in health-related research involving Indigenous peoples located in the U.S. and Canada.

Second, there is need for research to better understand how each of the components of data management contribute to a context in which Indigenous peoples in the U.S. and Canada are amenable to and able to benefit from participation in research. In particular, there is need for a more complete account of the conditions of data management that encourage Indigenous people to participate in research involving data sharing, genetic data, and other issues that heighten concerns related to risks of individual and group harm and stigma or where the preferences of Indigenous communities may be in conflict with conventional scientific practice. For example, our study reveals strong support for Indigenous community oversight over secondary uses of data; yet, federal or other institutional biobanks and data repositories frequently utilize in-house data access committees that are primarily staffed by scientific experts to determine who will have access to stored samples or data and for what research purposes. Research is needed to determine how such conventional practices might be modified to incorporate community perspectives and address community preferences and needs.

Third, there is need for standards for reporting on data management in the scientific literature and for data management tools—including research policies and protocols, memorandum of understanding, research codes, and data sharing agreements—that have been developed for and/or used in health-related research involving Indigenous people. Where such tools exist, there is need for making them accessible to communities and researchers through supplementary materials published concurrently with research articles; websites of professional, advocacy, and community groups; online clearinghouses for documents on project management; and, other venues. Where such tools are lacking, there is need for research to develop and test the feasibility, acceptability, and effectiveness of such tools.

### Strengths and Limitations

This review has several strengths. It employed a multi-stage screening process guided by formal protocols that allowed for efficient identification of relevant articles and involved independent review of each article by two authors. The data extraction form benefitted from the use of standardized response sets and notes and inclusion/exclusion criteria for all questions and from an iterative approach to development involving all authors.

This review also has limitations. Articles were extracted by one rather than by two reviewers. Although this increases the efficiency of the extraction process, it also prevents the identification of errors related to data interpretation and entry through secondary review. To account for this, two authors extracted one article using both the detailed and limited data extraction forms, to assess and promote the reliability of the data extraction process. The search strategy included four large databases; however, these databases are not inclusive of all articles. Future reviews should consider including Indigenous journals and databases maintained by Indigenous governance bodies. The small number of articles included in this review limit the ability to generalize findings to other Indigenous communities or health research studies.

## Conclusion

This scoping review sought to describe the terms of data management in health-related research involving Indigenous communities in the U.S. and Canada. We identified a limited number of articles that included detailed accounts of the terms of data management in this research context. Reviewed articles suggest that Indigenous communities in the U.S. and Canada have a stake in all components and principles of data management and that community engagement in and regulation of research can support the development and implementation of data management terms that respect the beliefs and align with the interests and values of Indigenous communities. These findings suggest that researchers conducting health-related participatory research with Indigenous peoples in the U.S. and Canada should proactively involve participating communities in decisions related to management of research data. Findings also suggest that the data management policies and practices actually employed in such research should be more fully reported in the peer-reviewed literature.

## Data Availability Statement

The datasets generated for this study are available on request to the corresponding author.

## Author Contributions

RW, JB, VH, and WB contributed to the development of the manuscript outline and text and the data extraction form. RW developed the screening protocols and search strategy. RW and JB conducted the screening protocol. RW and WB reviewed articles selected for inclusion and conducted data extraction.

## Funding

Research for this manuscript was funded by the National Institute of General Medical Sciences (grant number: 5P01GM116691) and the National Human Genome Research Institute (grant number: 5RM1HG009042). Funding agencies covered the cost of research staff time.

## Conflict of Interest

The authors declare that the research was conducted in the absence of any commercial or financial relationships that could be construed as a potential conflict of interest.
