# Supplementary material for: Data Management in Health-Related Research Involving Indigenous Communities in the United States and Canada: A Scoping Review
Source: Front Genet. 2019 Oct 10;10:942. doi: 10.3389/fgene.2019.00942 (PMC6796238; doi:10.3389/fgene.2019.00942)
Supplement: Supplementary file 1 [file Table_1.docx]

**Supplement 1: Search Strategy**

((((((((((((((((((("harm") AND ((((((((("indians, north american"[MeSH Major Topic]) OR "inuits"[MeSH Major Topic]) OR "alaska natives"[MeSH Major Topic]))))))))) OR (("research ethics") AND (((((((((("indians, north american"[MeSH Major Topic]) OR "inuits"[MeSH Major Topic]) OR "alaska natives"[MeSH Major Topic])))))))))))) OR (((((((((("data sharing" OR "data-sharing" OR "data use" OR "data management" OR "data control" OR "data governance" OR "data stewardship" OR "data ownership" OR "data harmonization" OR "data access" OR "data security" OR "data storage"))) AND (("process*" OR "document*" OR "record*" OR "standard*" OR "policy" OR "policies" OR "protocol" OR "protocols" OR "practice" OR "practices" OR "guideline" OR "guidelines" OR "recommendation" OR "recommendations" OR "principle" OR "principles"))) AND (("indians, north american"[MeSH Major Topic]) OR "inuits"[MeSH Major Topic]) OR "alaska natives"[MeSH Major Topic]))))))))) OR (((((((("colorectal cancer screening") AND ((((("indians, north american"[MeSH Major Topic]) OR "inuits"[MeSH Major Topic]) OR "alaska natives"[MeSH Major Topic]))))) OR ((("screening" AND "evaluation" AND "women" AND "nation")) AND ((((("indians, north american"[MeSH Major Topic]) OR "inuits"[MeSH Major Topic]) OR "alaska natives"[MeSH Major Topic]))))) OR (("special diabetes program") AND ((((("indians, north american"[MeSH Major Topic]) OR "inuits"[MeSH Major Topic]) OR "alaska natives"[MeSH Major Topic]))))) OR (("Cervical Cancer Early Detection Program") AND ((((("indians, north american"[MeSH Major Topic]) OR "inuits"[MeSH Major Topic]) OR "alaska natives"[MeSH Major Topic]))))) OR (("NBCCEDP") AND ((((("indians, north american"[MeSH Major Topic]) OR "inuits"[MeSH Major Topic]) OR "alaska natives"[MeSH Major Topic]))))))) OR ((("secondary research") AND ((((("indians, north american"[MeSH Major Topic]) OR "inuits"[MeSH Major Topic]) OR "alaska natives"[MeSH Major Topic])))))) OR ((((("withdrawal") AND ((((("indians, north american"[MeSH Major Topic]) OR "inuits"[MeSH Major Topic]) OR "alaska natives"[MeSH Major Topic]))))) OR (("consent process") AND ((((("indians, north american"[MeSH Major Topic]) OR "inuits"[MeSH Major Topic]) OR "alaska natives"[MeSH Major Topic]))))))) OR ((("identifier*") AND ((((((((("indians, north american"[MeSH Major Topic]) OR "inuits"[MeSH Major Topic]) OR "alaska natives"[MeSH Major Topic])))))))))) OR (((((((((("data sharing" OR "data-sharing")) AND ((((("indians, north american"[MeSH Major Topic]) OR "inuits"[MeSH Major Topic]) OR "alaska natives"[MeSH Major Topic]))))) OR (("data ownership") AND ((((("indians, north american"[MeSH Major Topic]) OR "inuits"[MeSH Major Topic]) OR "alaska natives"[MeSH Major Topic]))))) OR (("data access committee") AND ((((("indians, north american"[MeSH Major Topic]) OR "inuits"[MeSH Major Topic]) OR "alaska natives"[MeSH Major Topic]))))) OR (("data access") AND ((((((((("indians, north american"[MeSH Major Topic]) OR "inuits"[MeSH Major Topic]) OR "alaska natives"[MeSH Major Topic]))))))))) OR (("data repository") AND ((((((((("indians, north american"[MeSH Major Topic]) OR "inuits"[MeSH Major Topic]) OR "alaska natives"[MeSH Major Topic]))))))))) OR (("data management") AND ((((((((("indians, north american"[MeSH Major Topic]) OR "inuits"[MeSH Major Topic]) OR "alaska natives"[MeSH Major Topic]))))))))))) OR ((("Trust"[Mesh]) AND ((((((((("indians, north american"[MeSH Major Topic]) OR "inuits"[MeSH Major Topic]) OR "alaska natives"[MeSH Major Topic])))))))))) OR (((((((((((((((((((("Guideline" [Publication Type]) AND ((((((("indians, north american"[MeSH Major Topic]) OR "inuits"[MeSH Major Topic]) OR "alaska natives"[MeSH Major Topic]))))))) OR (("Guidelines as Topic"[Majr]) AND ((((((("indians, north american"[MeSH Major Topic]) OR "inuits"[MeSH Major Topic]) OR "alaska natives"[MeSH Major Topic]))))))) OR (((((((((("indians, north american"[MeSH Major Topic]) OR "inuits"[MeSH Major Topic]) OR "alaska natives"[MeSH Major Topic]))))))) AND "Practice Guidelines as Topic"[Majr])) OR (("Clinical Protocols"[Majr]) AND ((((((("indians, north american"[MeSH Major Topic]) OR "inuits"[MeSH Major Topic]) OR "alaska natives"[MeSH Major Topic]))))))) OR (((( "Clinical Protocols/history"[Mesh] OR "Clinical Protocols/methods"[Mesh] OR "Clinical Protocols/organization and administration"[Mesh] OR "Clinical Protocols/standards"[Mesh] OR "Clinical Protocols/statistics and numerical data"[Mesh] ))) AND (((((((("indians, north american"[MeSH Major Topic]) OR "inuits"[MeSH Major Topic]) OR "alaska natives"[MeSH Major Topic])))))))) OR (("Guideline Adherence"[Majr]) AND (((((((("indians, north american"[MeSH Major Topic]) OR "inuits"[MeSH Major Topic]) OR "alaska natives"[MeSH Major Topic])))))))) OR (((( "Guideline Adherence/ethics"[Mesh] OR "Guideline Adherence/history"[Mesh] OR "Guideline Adherence/legislation and jurisprudence"[Mesh] OR "Guideline Adherence/organization and administration"[Mesh] OR "Guideline Adherence/standards"[Mesh] OR "Guideline Adherence/statistics and numerical data"[Mesh] OR "Guideline Adherence/trends"[Mesh] OR "Guideline Adherence/utilization"[Mesh] ))) AND (((((((("indians, north american"[MeSH Major Topic]) OR "inuits"[MeSH Major Topic]) OR "alaska natives"[MeSH Major Topic])))))))) OR (("Records as Topic"[Majr]) AND (((((((("indians, north american"[MeSH Major Topic]) OR "inuits"[MeSH Major Topic]) OR "alaska natives"[MeSH Major Topic])))))))) OR (((( "Records as Topic/ethics"[Mesh] OR "Records as Topic/history"[Mesh] OR "Records as Topic/legislation and jurisprudence"[Mesh] OR "Records as Topic/methods"[Mesh] OR "Records as Topic/organization and administration"[Mesh] OR "Records as Topic/standards"[Mesh] OR "Records as Topic/statistics and numerical data"[Mesh] OR "Records as Topic/trends"[Mesh] OR "Records as Topic/utilization"[Mesh] ))) AND (((((((("indians, north american"[MeSH Major Topic]) OR "inuits"[MeSH Major Topic]) OR "alaska natives"[MeSH Major Topic])))))))) OR (("Policy Making"[Majr]) AND (((((((("indians, north american"[MeSH Major Topic]) OR "inuits"[MeSH Major Topic]) OR "alaska natives"[MeSH Major Topic])))))))) OR (((( "Policy Making/ethics"[Mesh] OR "Policy Making/history"[Mesh] OR "Policy Making/legislation and jurisprudence"[Mesh] OR "Policy Making/methods"[Mesh] OR "Policy Making/organization and administration"[Mesh] OR "Policy Making/standards"[Mesh] OR "Policy Making/statistics and numerical data"[Mesh] OR "Policy Making/trends"[Mesh] OR "Policy Making/utilization"[Mesh] ))) AND (((((((("indians, north american"[MeSH Major Topic]) OR "inuits"[MeSH Major Topic]) OR "alaska natives"[MeSH Major Topic])))))))) OR ((((((((((("indians, north american"[MeSH Major Topic]) OR "inuits"[MeSH Major Topic]) OR "alaska natives"[MeSH Major Topic])))))))) AND "Decision Making, Organizational"[Mesh])) OR (("Program Development"[Majr]) AND ((((((((("indians, north american"[MeSH Major Topic]) OR "inuits"[MeSH Major Topic]) OR "alaska natives"[MeSH Major Topic]))))))))) OR (((( "Program Development/methods"[Mesh] OR "Program Development/organization and administration"[Mesh] OR "Program Development/standards"[Mesh] OR "Program Development/statistics and numerical data"[Mesh] ))) AND ((((((((("indians, north american"[MeSH Major Topic]) OR "inuits"[MeSH Major Topic]) OR "alaska natives"[MeSH Major Topic]))))))))) OR (("Program Evaluation"[Majr]) AND ((((((((("indians, north american"[MeSH Major Topic]) OR "inuits"[MeSH Major Topic]) OR "alaska natives"[MeSH Major Topic]))))))))) OR (((( "Program Evaluation/ethics"[Mesh] OR "Program Evaluation/history"[Mesh] OR "Program Evaluation/legislation and jurisprudence"[Mesh] OR "Program Evaluation/methods"[Mesh] OR "Program Evaluation/organization and administration"[Mesh] OR "Program Evaluation/standards"[Mesh] OR "Program Evaluation/statistics and numerical data"[Mesh] OR "Program Evaluation/trends"[Mesh] OR "Program Evaluation/utilization"[Mesh] ))) AND ((((((((("indians, north american"[MeSH Major Topic]) OR "inuits"[MeSH Major Topic]) OR "alaska natives"[MeSH Major Topic]))))))))) OR ((((((((((("indians, north american"[MeSH Major Topic]) OR "inuits"[MeSH Major Topic]) OR "alaska natives"[MeSH Major Topic])))))))) AND "Organizational Policy"[Majr])))) OR ((((( "Genetics/ethics"[Mesh] OR "Genetics/history"[Mesh] OR "Genetics/legislation and jurisprudence"[Mesh] OR "Genetics/methods"[Mesh] OR "Genetics/organization and administration"[Mesh] OR "Genetics/standards"[Mesh] OR "Genetics/statistics and numerical data"[Mesh] OR "Genetics/trends"[Mesh] OR "Genetics/utilization"[Mesh] ))) AND (((((("indians, north american"[MeSH Major Topic]) OR "inuits"[MeSH Major Topic]) OR "alaska natives"[MeSH Major Topic]))))))) OR (((((((((((((((((("indians, north american"[MeSH Major Topic]) OR "inuits"[MeSH Major Topic]) OR "alaska natives"[MeSH Major Topic]))))) AND "Pharmacogenetics"[Majr])) OR (((( "Pharmacogenetics/ethics"[Mesh] OR "Pharmacogenetics/history"[Mesh] OR "Pharmacogenetics/legislation and jurisprudence"[Mesh] OR "Pharmacogenetics/methods"[Mesh] OR "Pharmacogenetics/organization and administration"[Mesh] OR "Pharmacogenetics/standards"[Mesh] OR "Pharmacogenetics/statistics and numerical data"[Mesh] OR "Pharmacogenetics/trends"[Mesh] ))) AND (((((("indians, north american"[MeSH Major Topic]) OR "inuits"[MeSH Major Topic]) OR "alaska natives"[MeSH Major Topic])))))) OR (("Genetic Research"[Majr]) AND (((((("indians, north american"[MeSH Major Topic]) OR "inuits"[MeSH Major Topic]) OR "alaska natives"[MeSH Major Topic])))))) OR (((( "Genetic Research/ethics"[Mesh] OR "Genetic Research/history"[Mesh] OR "Genetic Research/legislation and jurisprudence"[Mesh] OR "Genetic Research/organization and administration"[Mesh] OR "Genetic Research/statistics and numerical data"[Mesh] OR "Genetic Research/trends"[Mesh] ))) AND (((((("indians, north american"[MeSH Major Topic]) OR "inuits"[MeSH Major Topic]) OR "alaska natives"[MeSH Major Topic])))))) OR (("Genetics, Medical"[Majr]) AND (((((("indians, north american"[MeSH Major Topic]) OR "inuits"[MeSH Major Topic]) OR "alaska natives"[MeSH Major Topic])))))) OR (((( "Genetics, Medical/ethics"[Mesh] OR "Genetics, Medical/history"[Mesh] OR "Genetics, Medical/legislation and jurisprudence"[Mesh] OR "Genetics, Medical/methods"[Mesh] OR "Genetics, Medical/organization and administration"[Mesh] OR "Genetics, Medical/standards"[Mesh] OR "Genetics, Medical/statistics and numerical data"[Mesh] OR "Genetics, Medical/trends"[Mesh] OR "Genetics, Medical/utilization"[Mesh] ))) AND (((((("indians, north american"[MeSH Major Topic]) OR "inuits"[MeSH Major Topic]) OR "alaska natives"[MeSH Major Topic])))))) OR (("genetic privacy"[MeSH Terms]) AND (((((("indians, north american"[MeSH Major Topic]) OR "inuits"[MeSH Major Topic]) OR "alaska natives"[MeSH Major Topic])))))) OR (("Databases, Genetic"[Majr]) AND ((((((("indians, north american"[MeSH Major Topic]) OR "inuits"[MeSH Major Topic]) OR "alaska natives"[MeSH Major Topic]))))))) OR (((( "Databases, Genetic/ethics"[Majr] OR "Databases, Genetic/history"[Majr] OR "Databases, Genetic/legislation and jurisprudence"[Majr] OR "Databases, Genetic/organization and administration"[Majr] OR "Databases, Genetic/standards"[Majr] OR "Databases, Genetic/statistics and numerical data"[Majr] OR "Databases, Genetic/trends"[Majr] OR "Databases, Genetic/utilization"[Majr] ))) AND ((((((("indians, north american"[MeSH Major Topic]) OR "inuits"[MeSH Major Topic]) OR "alaska natives"[MeSH Major Topic]))))))))) OR (((((("indians, north american"[MeSH Major Topic]) OR "inuits"[MeSH Major Topic]) OR "alaska natives"[MeSH Major Topic])) AND "Incidental Findings"[Mesh:NoExp]))) OR (((((((("Informed Consent"[Majr]) AND ((("indians, north american"[MeSH Major Topic]) OR "inuits"[MeSH Major Topic]) OR "alaska natives"[MeSH Major Topic]))) OR (((( "Informed Consent/ethics"[Mesh] OR "Informed Consent/history"[Mesh] OR "Informed Consent/legislation and jurisprudence"[Mesh] OR "Informed Consent/methods"[Mesh] OR "Informed Consent/organization and administration"[Mesh] OR "Informed Consent/standards"[Mesh] OR "Informed Consent/statistics and numerical data"[Mesh] OR "Informed Consent/trends"[Mesh] OR "Informed Consent/utilization"[Mesh] ))) AND ((("indians, north american"[MeSH Major Topic]) OR "inuits"[MeSH Major Topic]) OR "alaska natives"[MeSH Major Topic]))) OR (((((("indians, north american"[MeSH Major Topic]) OR "inuits"[MeSH Major Topic]) OR "alaska natives"[MeSH Major Topic]))) AND "Consent Forms"[Majr])) OR (("Freedom"[Majr]) AND (((("indians, north american"[MeSH Major Topic]) OR "inuits"[MeSH Major Topic]) OR "alaska natives"[MeSH Major Topic])))) OR (("Freedom"[Mesh:NoExp]) AND (((("indians, north american"[MeSH Major Topic]) OR "inuits"[MeSH Major Topic]) OR "alaska natives"[MeSH Major Topic])))))) OR ((((((("Confidentiality"[Majr]) AND (((("Indians, North American"[Majr]) OR "Alaska Natives"[Majr]) OR "Inuits"[Majr])))) OR (((( "Confidentiality/ethics"[Mesh] OR "Confidentiality/history"[Mesh] OR "Confidentiality/legislation and jurisprudence"[Mesh] OR "Confidentiality/organization and administration"[Mesh] OR "Confidentiality/standards"[Mesh] OR "Confidentiality/statistics and numerical data"[Mesh] OR "Confidentiality/trends"[Mesh] ))) AND (((("Indians, North American"[Majr]) OR "Alaska Natives"[Majr]) OR "Inuits"[Majr])))) OR (("Privacy"[Majr]) AND (((("Indians, North American"[Majr]) OR "Alaska Natives"[Majr]) OR "Inuits"[Majr])))) OR (((( "Privacy/ethics"[Mesh] OR "Privacy/history"[Mesh] OR "Privacy/legislation and jurisprudence"[Mesh] OR "Privacy/organization and administration"[Mesh] OR "Privacy/standards"[Mesh] OR "Privacy/statistics and numerical data"[Mesh] OR "Privacy/trends"[Mesh] ))) AND (((("Indians, North American"[Majr]) OR "Alaska Natives"[Majr]) OR "Inuits"[Majr])))))) OR ((((((((((("Information Storage and Retrieval"[Majr])) AND ((("Indians, North American"[Majr]) OR "Alaska Natives"[Majr]) OR "Inuits"[Majr]))) OR (((( "Information Storage and Retrieval/ethics"[Mesh] OR "Information Storage and Retrieval/history"[Mesh] OR "Information Storage and Retrieval/legislation and jurisprudence"[Mesh] OR "Information Storage and Retrieval/methods"[Mesh] OR "Information Storage and Retrieval/organization and administration"[Mesh] OR "Information Storage and Retrieval/standards"[Mesh] OR "Information Storage and Retrieval/statistics and numerical data"[Mesh] OR "Information Storage and Retrieval/trends"[Mesh] OR "Information Storage and Retrieval/utilization"[Mesh] ))) AND ((("Indians, North American"[Majr]) OR "Alaska Natives"[Majr]) OR "Inuits"[Majr]))) OR (("computer security"[MeSH Terms]) AND (((((("indians, north american"[MeSH Major Topic]) OR "inuits"[MeSH Major Topic]) OR "alaska natives"[MeSH Major Topic])))))) OR (((((((("indians, north american"[MeSH Major Topic]) OR "inuits"[MeSH Major Topic]) OR "alaska natives"[MeSH Major Topic]))))) AND "Databases as Topic"[Majr])) OR (((( "Databases as Topic/ethics"[Mesh] OR "Databases as Topic/history"[Mesh] OR "Databases as Topic/legislation and jurisprudence"[Mesh] OR "Databases as Topic/organization and administration"[Mesh] OR "Databases as Topic/standards"[Mesh] OR "Databases as Topic/statistics and numerical data"[Mesh] OR "Databases as Topic/trends"[Mesh] OR "Databases as Topic/utilization"[Mesh] ))) AND (((((("indians, north american"[MeSH Major Topic]) OR "inuits"[MeSH Major Topic]) OR "alaska natives"[MeSH Major Topic])))))) OR (("Information Dissemination"[Majr]) AND ((((("Indians, North American"[Majr]) OR "Alaska Natives"[Majr]) OR "Inuits"[Majr]))))) OR (((( "Information Dissemination/ethics"[Mesh] OR "Information Dissemination/history"[Mesh] OR "Information Dissemination/legislation and jurisprudence"[Mesh] OR "Information Dissemination/methods"[Mesh] OR "Information Dissemination/organization and administration"[Mesh] ))) AND ((((("Indians, North American"[Majr]) OR "Alaska Natives"[Majr]) OR "Inuits"[Majr]))))))
