# Supplementary material for: Data Management in Health-Related Research Involving Indigenous Communities in the United States and Canada: A Scoping Review
Source: Front Genet. 2019 Oct 10;10:942. doi: 10.3389/fgene.2019.00942 (PMC6796238; doi:10.3389/fgene.2019.00942)
Supplement: Supplementary file 2 [file Table_2.docx]

**Supplement 2: Article Screening Protocol**

**Scoping Review: Filtering Protocol**

| **Box 1. EndNote Library Group-set and Group**   - - [Group-set] Database Libraries     - [Group] Database 1     - [Group] Database 2     - [Group] All Articles   - [Group-set] Duplicates     - Duplicates Identified by EndNote     - Duplicates Identified by Reviewer   - [Group-set] Reference Type     - [Group] Book + Book Section (Removed)     - [Group] Thesis (Removed)     - [Group] Conference Proceedings     - [Group] Other Reference Types     - [Group] Journal Article   - [Group-set] Title Review     - [Group] Full Title Review Library     - [Group] Category A1: Not Relevant     - [Group] Category A2: Relevant     - [Group] Category A3: Potentially Relevant   - [Group-set] Keyword and Rapid Abstract Review     - [Group] Full Keyword and Rapid Abstract Review Library     - [Group] Category B1: Not Relevant     - [Group] Category B2: Relevant     - [Group] Category B3: Background   - [Group-set] Relevant Articles: Combined + Discrepant     - [Group] Reviewer 1 Relevant Articles (Category A2 + Category B2)     - [Group] Reviewer 2 Relevant Articles (Category A2 + Category B2)     - [Group] Reviewer 1 + Reviewer 2 Relevant Articles (Category A2 + Category B2)     - [Group] Reviewer 1 + Reviewer 2 Relevant Articles (Discrepant Articles)     - [Group] Reviewer 1 + Reviewer 2 Relevant Articles (Non-Discrepant Articles)   - [Group-set] Background Articles: Combined + Discrepant     - [Group] Reviewer 1 Background Articles (Category B3)     - [Group] Reviewer 2 Background Articles (Category B3)     - [Group] Reviewer 1 + Reviewer 2 Background Articles (Category B3)     - [Group] Reviewer 1 + Reviewer 2 Background Articles (Discrepant Articles)     - [Group] Reviewer 1 + Reviewer 2 Background Articles (Non-Discrepant Articles)   - [Group-set] Discrepancy Review: Title Review     - [Group] Category A1: Not Relevant     - [Group] Category A2: Relevant     - [Group] Category A3: Potentially Relevant     - [Group] Full Title Review Library of Discrepant Articles   - [Group-set] Discrepancy Review: Keyword and Rapid Abstract Review     - [Group] Category B1: Not Relevant     - [Group] Category B2: Relevant     - [Group] Category B3: Background     - [Group] Full Keyword and Rapid Abstract Review Library of Discrepant Articles   - [Group-set] Discrepancy Review Results     - Background Articles (Category B3)     - Relevant Articles (Category A2 + Category B2)   - [Group-set] Final Results     - All Background Articles (Category B3)     - All Relevant Articles (Category A2 + Category B2) |
| --- |

**Step 1: Prepare the Library for Title-Filtering and Rapid Abstract Review**

- In EndNote, create the group-sets and groups listed in Box 1.
- Move articles from each database into the respective group under the “Database Libraries” group-set.
- Combine articles from all database libraries into the “All Articles” group under the “Database Libraries” group-set.
- Use the EndNote “Find Duplicates” Tool to identify duplicates references. Move duplicates to the “Duplicates Identified by EndNote” group.
- Sort references by type into the appropriate groups under the “Reference Type” group-set.
- Move references in the “Journal Article” group into the “Full Title Review Library” group under the “Title Review” group-set. [Note: For convenience, these references will subsequently be referred to as “articles”.]
- Sort articles in the “Working Library” group alphabetically by last name of first listed author.

**Step 2: Review and Filter Articles by Title**

Article relevance is determined by the presence of keyword combinations in the article title. Use the rules listed below and the keywords provided in Table 1 to sort articles.

- Filter all articles in the “Title Review Library” by Sort articles into one of three categories: Category A1—articles that are not relevant to the scoping review; Category A2—articles that are relevant to the scoping review; and, Category A3—articles of uncertain relevance to the scoping review.
  - Category A2: Article titles include keywords from: (1) Groups A, B, and C; (2) Groups A, B, and D; or, (3) Group E.
  - Category A3: Article titles include keywords from: (1) Groups A and C; (2) Groups B and C; (3) Groups B and D; (4) Groups C and D; or, (5) Groups A and D.2.
  - Category A1: Article titles that do not meet the requirements for Category A2 or A3.

| **Table 1. Keyword Groups** | |
| --- | --- |
| **Group A: Population Keywords** | Population Keywords: Native American, American Indian, Alaska Native, Indigenous, Aboriginal, First Peoples, tribes/tribal, specific AN/AI groups, and related terms. |
| **Group B: Document Keywords** | Document Keywords: Policy, Policies, protocol(s), procedure(s), standard(s), guideline(s), practice(s), process(es), agreements, forms, governance, and related terms. |
| **Group C: Major Topic Keywords** | C.1 Data Use Keywords: data-sharing, data management, data control, data security, data ownership, data stewardship, data governance, data collection, data storage, data anonymization, data release, data use, database, biobank, data repository, biospecimen, and related terms. |
|  | C.2 ESLI Keywords: autonomy, withdrawal, incidental findings, secondary research, return of results, privacy, confidentiality, re-identification, de-identification, anonymization, consent, ethics/ethical, stigma, trust/mistrust, participant/patient/community rights, informed consent, and related terms. |
|  | C.3 Research Process Keywords: data access committee, institutional review board, tribal review, research codes, dissemination, and related terms. |
| **Group D: Minor Topic Keywords** | D.1 Participatory Research Keywords: community-academic relationships/partnerships, community perspectives, community engagement, community-based participatory research, community advisory board, culturally appropriate/sensitive/adapted, and related terms. |
|  | D.2 Genetic Research Keywords: genes, DNA, genetics, genomics, epigenetics, genome, exome, sequencing, pharmacogenetics, and related terms. |
| **Group E: Program Keywords** | Program Keywords: Wisewoman, special diabetes program, colorectal cancer screening program, cervical cancer early detection program, NBCCEDP, and human genome project. [Note: Group E keywords include only those terms listed here.] |

**Step 3: Review and Filter Articles by Keyword**

Articles moved to Category A3 must be sorted by relevance into Category B1, B2, or B3. Article relevance is determined by the presence of keyword combinations in the Keyword Section listed on the article information page. Use the rules listed below and the keywords provided in Table 1 to sort articles.

- Filter all articles in the “Keyword Review Library” by reviewing the Keyword Section listed on the article information page.
  - Articles with a Keyword Section that lists keywords from: (1) Groups A, B, and C; (2) Groups A, B, and D; (3) Group E are moved to Category B2.
  - Articles with a Keyword Section that lists keywords from: (1) Groups A and C; (2) Groups B and C; (3) Groups B and D; (4) Groups C and D; or, (5) Groups A and D.2 are moved to Category B3.
  - Articles with a Keyword Section that does not meet the requirements for Category B1 or rapid abstract review are moved to Category B1.

**Step 4: Review and Filter Articles by Abstract**

Articles filtered into Move these articles to the “Full Abstract Review Library” group under the “Abstract Review” group-set.

- Filter all articles in the “Abstract Review Library” by reviewing the abstract listed on the article information page.
  - Articles with an abstract that lists keywords from: (1) Groups A, B, and C; (2) Groups A, B, and D; or (3) Group E are moved to Category C2.
  - Articles with an abstract that lists keywords from: (1) groups A, C.2, and C.1; or, (2) groups A, C.2, and C.3 are moved to Category C3.
  - Articles with an abstract that does not meet requirements for Category 3A or 3B are moved to Category C1.

[Note: Category A3 articles that lack keywords but have an abstract are reviewed as described in Step 4. Reviewers must locate abstracts for Category 3 articles that lack an abstract and that require a rapid abstract review.]

**Step 4: Combine All Relevant Articles**

- After completing title-filtering process, combine articles from Category A2 and Category B2 into the “Reviewer X Relevant Articles (Category A2 + Category B2)” group under the “Relevant Articles: Combined + Discrepant” group-set. Move the articles in Category B3 to the “Reviewer X Background Articles (Category B3)” under the “Background Articles: Combined + Discrepant” group-set.
- If you are responsible for conducting the discrepancy review, continue to Step 5. If you are not conducting the discrepancy review, provide your library to the individual who is.

**Step 5: Conduct Discrepancy Review**

- Combine articles from the “Reviewer 1 + Reviewer 2 Relevant Articles (Discrepant Articles)” and the “Reviewer 1 + Reviewer 2 Background Articles (Discrepant Articles)” groups in the “Full Title Review Library of Discrepant Articles” under the “Discrepancy Review: Title Review” group-set.
- Repeat steps 2-3. Filter articles into the appropriate groups under the “Discrepancy Review: Title Review” group-set and then the “Discrepancy Review: Keyword and Rapid Abstract Review” group-set.
- Move relevant and background articles from discrepancy review into the appropriate group under the “Discrepancy Review Results” group-set.
- Combine relevant and background articles from the discrepancy review with the non-discrepant results from original review into the appropriate groups under the “Final Results” group-set.

**Additional Notes**

- Move duplicate articles identified during the title-filtering process to the “Duplicate Identified by Reviewer during Title-Filter” group.
- Reviewers will beta-test the title-filtering protocol by applying the protocol to the first 100 articles listed alphabetically by last name of first author.
- Reviewers must use their judgment if they come across an article that has potential relevance for the research questions, but does not meeting the requirements for inclusion in groups A2, B2, or B3.
