# Supplementary material for: Data Management in Health-Related Research Involving Indigenous Communities in the United States and Canada: A Scoping Review
Source: Front Genet. 2019 Oct 10;10:942. doi: 10.3389/fgene.2019.00942 (PMC6796238; doi:10.3389/fgene.2019.00942)
Supplement: Supplementary file 3 [file Table_3.docx]

**Supplement 3: Detailed Data Extraction Form**

| **Query #** | **Query** | **Response Types** | **Notes & Inclusion/Exclusion Criteria** |
| --- | --- | --- | --- |
| 1 | Who is reviewing this article/document? | Open | [Note: Provide reviewer initials.] |
| 2 | What is the last name of the first listed author? | Open | [Note: Provide the last name only of the first listed author.] |
| 3 | What is the year of publication of the article/document? | Open | [Note: Provide the date of publication, **not** the date of acceptance. For articles with multiple publication dates (e.g., online, in print), use the earliest publication date.] |
| 4 | Who funded this research? | Open 0=NR | [Note: If funder is identified, provide the name of the funding organization. List specific NIH IC if available. Use "NR" if the funder is not reported.] |
| 5 | If this is primary/original research, provide the study design and key methods of data collection. | 0=RCT 1=Cohort study 2=Case-control study 3=Cross-sectional study 4=Case study/Case Series 5=Description/Evaluation of Program Process/Outcomes 6=Other Study Design 7=NR 8=NA | [Note: Use "NR" if the study design is unknown. Use "NA" if the article is not primary/original research.] |
| 6 | If this is primary/original research, what data collection methods were used? | 0=Interview 1=Focus Group 2=Survey 3=Other Data Collection Methods 4=NR 5=NA | [Note: Use "NR" if methods are unknown. Use "NA" if the article is not primary/original research.] |
| 7 | If this is a literature review, please indicate the type of review. | 0=Systematic review 1=Meta-analysis 2=Other type of literature review 3=NR 4=NA | [Note: Provide only one response. Use "NR" if the type of review is unknown. Use "NA" if the article is not a literature review.] |
| 8 | Was (were) the research project(s) or program(s) described as employing a specific or a general participatory research approach? | 0=Specific participatory research approach 1=General participatory research approach 2=Non-participatory research approach 3=NR | [Note: Use "specific" if the study or project/program is explicitly described as employing a specific participatory methodology (e.g., CBPR, TPR, PAR). Use "general" if the study or project/program is described as involving community engagement/participation/empowerment or a similar term, but does not explicitly describe use of a particular participatory research approach. Use "non-participatory" if a non-participatory approach is described. Use "NR" if the approach is unknown.] |
| 9 | Provide the article purpose and/or project/study purpose or research question. | Open | [Note: Describe the purpose of the article. If the article primarily concerns a program or research study, provide the project/program's purpose or the research study's research question. If the article/study purpose is provided, quote it directly. Place direct quotes in quotation marks.] |
| 10 | List the health condition or issue that is addressed by the program or study described in the article. | Open 0=NA | [Note: Limit response to 1-2 words (e.g., "diabetes", "cancer"). Use "NA" if this is a program/study that does not target a specific health condition or set of health conditions.] |
| 11 | In what state do the program/study activities described in the article take place? | Open 0=NR | [Note: If available, provide the two-letter abbreviation of all states in which the population/community involved in the program/study is located. research is located. Separate multiple state abbreviations with commas. If only the region (e.g., pacific northwest, southwest) is described, provide the region. Use "NR" if the state/region is not provided.] |
| 12 | Does the program/study take place in a community located on a federally recognized reservation? | 0=Yes 1=No 2=NR | [Note: Use "NR" if it is unknown whether the research takes place on a federally recognized reservation.] |
| 13 | Briefly describe any challenges, issues, or limitations related to data management. | Open 0=NR | [Note: Paraphrase or use direct quotes. Place direct quotes in quotation marks. Only list those limitations, issues, or challenges that are explicitly described in the article and that are related to data management. Use "NR" if no challenges, issues, or limitations related to data management are described.] |
| 14 | Does the article discuss data management or components of data management, including: data collection, storage, security, use, sharing, ownership, control, access, possession; databases/biobanks; data management tools; and, results dissemination. | 0=Yes 1=No |  |
| 15 | Does the article describe the use or development of any data management tools? | 0=Y 1=N | [Note: Data management tools include policies/protocols, groups, and systems/databases.] |
| 16 | If you answered "Y" to query #15, was the community involved in the development or selection of the data management tools that were developed? | 0=Yes 1=No 2=NR 3=NA | [Note: Use "NR" if it is unknown whether the community was involved in development of data management protocols/policies. Use "NA" if you responded "N" to query #15.] |
| 17 | If you answered "Y" to query #15, indicate whether the tool involves or addresses data collection, data sharing, ownership/control of data, data storage/security, dissemination or return of results, and/or conditions for withdrawal of data. | 0=Data Collection 1=Data sharing 2=Data ownership/control 3=Data storage/security 4=Dissemination and/or return of results 5=Data withdrawal/disposal 6=Other 7=NR 8=NA 9=Data Analysis/Interpretation 10=Data Ownership 11=Data Control 12=Data Access 13=Data Possession | [Note: Use "Other" if the protocol/policy addresses a component of data management not covered under other standardized responses. Use "NR" if details on the data management tool are unknown. Use "NA" if the article response to query #15 was "N".] |
| 18 | Did the research study or project/program collect biospecimens? | 0=Yes 1=No 2=NR | [Note: Use "Y" if the study or project/program does collect biospecimens. Use "N" if the study does not collect biospecimens. Use "NR" if it is unknown whether the study or project/program collected biospecimens.] |
| 19 | Did the research study or project/program collect genetic data? | 0=Yes 1=No 2=NR | [Note: "NR" if it is unknown whether genetic data was collected.] |
| 20 | If you answered "Y" to query #19, describe the type of genetic data collected. | Open 0=NR 1=NA | [Note: Use "NA" if you did not answer "Y" to query #19. Use "NR" if genetic data was collected, but the type of genetic data collected is unknown.] |
| 21 | Does the study or project/program collect data other than biospecimens, genetic data, or demographic data? | 0=Yes 1=No 2=NR | [Note: Do not report collection of demographic information. Use "NR" if it is unknown whether types of data other than biospecimens or genetic or demographic information were collected.] |
| 22 | If you answered "Y" to query #21, describe the type of data collected. | Open 0=NR 1=NA | [Note: Use "NR" if the other type(s) of data collected is unknown. Use "NA" if you did not answer "Y" to query #21.] |
| 23 | Does the article describe processes used to analyze or interpret data or results or the terms of data analysis/interpretation? | 0=Y 1=N |  |
| 24 | If you answered "Y" to query 23, briefly paraphrase or directly quote the processes used to analysis or interpret data or results. | Open 0=NA | [Note: Place direct quotes in quotation marks.] |
| 25 | Were technical, administrative, physical, or other security measures taken to secure participant data? | 0=Yes 1=No 2=NR 3=NA | [Note: Use "NR" if it is not known whether security measures were taken to secure data. Use "NA" if no participant data was collected.] |
| 26 | If you answered "Y" to query #25, briefly describe the security measures. | 0=Technical measures 1=Administrative measures 2=Physical measures 3=Other measures 4=NR 5=NA | [Note: Use "NA" if you did not answer "Y" to query #25. Use "NR" if security measures were taken but are not described. Technical security measures include encrypted files or email and password protected computers and files. Administrative security measures include limits on the personnel who can access data and processes for disposing/storing data. Physical security measures include locked file cabinets and card-access offices. If using "Other", provide a brief description.] |
| 27 | Briefly describe the method(s) used to de-identify data collected for the study or project/program. | Open 0=NR 1=NA | [Note: Use "NR" if it is unknown whether deidentification methods were used or if deidentification methods were used but not described. Use "NA" if deidentification methods were not used.] |
| 28 | Is collected data stored in a public/private database/biobank? | 0=Yes 1=No 2=NR 3=NA | [Note: Use "NR" if it is not known whether data was shared in a public/private database. Use "NA" if the study or project/program did not involve collection of data that could be stored in a database/biobank.] |
| 29 | If you answered "Y" to query #28, indicate the type of data stored in the biobank/database. | Open 0=NR 1=NA | [Note: Use "NA" if you did not answer "Y" to query #28. Use "NR" if the type of data shared in a database is not described.] |
| 30 | If you answered "Y" to query #28, list the name of the database in the open response column. | Open 0=NR 1=NA | [Note: Provide the name of the database. Use "NA" if you did not answer "Y" to query #28. Use "NR" if the database name was not reported.] |
| 31 | Did the article describe any data sharing activities? | 0=Yes 1=No 2=NR | [Note: Use "NR" if it is unknown whether data sharing activities took place.] |
| 32 | If you answered "Y" to query #31, briefly describe these activities. | Open 0=NA | [Note: Use "NA" if you did not answer "Y" to query #31. For open responses, paraphrase or use direct quotes. Place direct quotes in quotation marks.] |
| 33 | Is sharing data for secondary research allowable? | 0=Yes 1=No 2=NR |  |
| 34 | If sharing data for secondary research is allowable, who approves the sharing of data for specific secondary research projects? | 0=Study Participants 1=Community Representatives 2=Academic researchers 3=Other 4=NA |  |
| 35 | Were study or project/program results/findings returned to participants or disseminated to community members? | 0=Yes 1=No 2=NR 3=NA | [Note: Use "Y" if results were available and returned to community members. Use "N" if results were available but were not returned to community members. Use "NR" if results were available but it is unknown whether they were shared with community members. Use "NA" if results were not available.] |
| 36 | If you answered "Y" to query #35, briefly describe the methods of return or dissemination (e.g., type of data shared, with whom the data was shared). | Open 0=NR 1=NA | [Note: Use "NR" if the methods of return/dissemination are not described. Use "NA" if you did not answer "Y" to query #35. Paraphrase or provide direct quotes when describing methods of results return/dissemination. Place direct quotes in quotation marks.] |
| 37 | Was the community involved in review and/or approval of publications/presentations of research findings? | 0=Yes 1=No 2=NR 3=NA |  |
| 38 | Did the community have veto power over whether publications/presentations of research findings were shared beyond the community? | 0=Yes 1=No 2=NR 3=NA |  |
| 39 | Did the article discuss processes related to the withdrawal of data from research or databases or the disposal or destruction of data? | 0=Yes 1=No 2=NR |  |
| 40 | If you answered "Y" to query #39, briefly describe the terms of data disposal or withdrawal. | Open 0=NA |  |
| 41 | Does the article discuss community-level governance of research? | 0=Yes 1=No |  |
| 42 | Did the community participating in research employ its own methods for guiding/regulating research (e.g., research codes, tribal resolution, CAB)? | 0=Yes 1=No 2=NR | [Note: Use "NR" if it is unknown whether the community employed its own methods of research regulation.] |
| 43 | If you answered "Y" to query #42, describe the method of research regulation. | 0=Tribal IRB 1=Research Code/Agreement 2=MOU/MOA 3=Tribal Resolution/Approval, 4=CAB Review/Approval,  5=Other 6=NR 7=NA |  |
| 44 | Did the community maintain ownership and/or control over data collected from/about the community or its members? | 0=Yes 1=No 2=NR 3=NA | [Note: Use "NR" if it is unknown whether the community maintained ownership and/or control over data. Use "NA" if no data was collected from the community or its members.] |
| 45 | If you answered "Y" to query #44, briefly describe the terms of ownership/control. | Open 0=NR 1=NA | [Note: Use "NA" if you did not answer "Y" to query #44. Use "NR" if details about the terms of ownership/control are not provided. Paraphrase or provide direct quotes. Place direct quotes in quotation marks.] |
| 46 | Does the article discussion the terms of data ownership, control, access, or possession? | 0=Ownership 1=Control 2=Access 3=Possession 4=No | [Note: Data ownership refers to the property status of data. Data control refers to the power to determine the fate of data, including whether how it is collected, used, and shared. Data access refers to the ability of individuals and groups to view their data and encompasses both access and accessibility. Data possession refers to the physical control over data.] |
| 47 | Did the community retain ownership, control, access to, or possession of data? | 0=ownership 1=control 2=access 3=possession 4=no |  |
| 48 | Did the community retain ownership of research data? | 0=Yes 1=No 2=NR 3=NA | [Note: Data ownership refers to the property status of data.] |
| 49 | If you answered "Y" to query #48, briefly describe the terms of data ownership. | Open 0=NA | [Note: Place direct quotes in quotation marks.] |
| 50 | Did the community retain control over research data? | 0=Yes 1=No 2=NR 3=NA | [Note: Data control refers to the power to determine the fate of data, including whether how it is collected, used, and shared.] |
| 51 | If you answered "Y" to query #50, briefly describe the terms of data control. | Open 0=NA | [Note: Place direct quotes in quotation marks.] |
| 52 | Did the community retain access to research data? | 0=Yes 1=No 2=NR 3=NA | [Note: Data access refers to the ability of individuals and groups to view their data and encompasses both access and accessibility.] |
| 53 | If you answered "Y" to query #52, briefly describe the terms of data access. | Open 0=NA | [Note: Place direct quotes in quotation marks.] |
| 54 | Did the community retain possession of data? | 0=Yes 1=No 2=NR 3=NA | [Note: Data possession refers to the physical control over data. Data is possessed by an individual or group when it is stored in facilities or on equipment owned and controlled by the individual or group.] |
| 55 | If you answered "Y" to query #54, briefly described the terms of data possession. | Open 0=NA | [Note: Place direct quotes in quotation marks.] |
| 56 | Did the article discuss community engagement in the research process? | 0=Yes 1=No 2=NR 3=NA |  |
| 57 | Was the community involved in activities or decisions related to data collection, data analysis and/or interpretation, data security and/or storage, data sharing, dissemination of research results, or withdrawal and/or disposal of data? | 0=Data Collection 1=Data Analysis/Interpretation 2=Data Storage/Security 3=Data Sharing 4=Dissemination/RoR 5=Withdrawal/Disposal 6=NR |  |
| 58 | If the community was involved in activities or decisions related to data collection, briefly paraphrase or directly quote this involvement. | Open 0=NA | [Note: Place direct quotes in quotation marks.] |
| 59 | If the community was involved in activities or decisions related to data analysis or interpretation, briefly paraphrase or directly quote this involvement. | Open 0=NA | [Note: Place direct quotes in quotation marks.] |
| 60 | If the community was involved in data security or storage, briefly paraphrase or directly quote this involvement. | Open 0=NA | [Note: Place direct quotes in quotation marks.] |
| 61 | If the community was involved in activities or decisions related to data sharing, briefly paraphrase or directly quote this involvement. | Open 0=NA | [Note: Place direct quotes in quotation marks.] |
| 62 | If the community was involved in activities or decisions related to dissemination/RoR, briefly paraphrase or directly quote this involvement. | Open 0=NA | [Note: Place direct quotes in quotation marks.] |
| 63 | If the community was involved in activities or decisions related to data withdrawal/disposal, briefly paraphrase or directly quote this involvement. | Open 0=NA | [Note: Place direct quotes in quotation marks.] |
| 64 | Did the article include any standards, guidelines, recommendations related to data management that are original to the article? | 0=Y 1=N |  |
| 65 | If you answered "Y" to query #64, briefly paraphrase or directly quote the standards, guidelines, or recommendations. | Open 0=NA | [Note: Place direct quotes in quotation marks. Do not describe specific data management protocols/policies listed in query #15. Use "NA" if you did not answer "Y" to query #65.] |
| 66 | Provide any information relevant to data management protocols/policies or activities that is not captured by the preceding queries. | Open | [Note: This query is optional. As appropriate, provide a response in the open response column.] |
