# Supplementary material for: Data Management in Health-Related Research Involving Indigenous Communities in the United States and Canada: A Scoping Review
Source: Front Genet. 2019 Oct 10;10:942. doi: 10.3389/fgene.2019.00942 (PMC6796238; doi:10.3389/fgene.2019.00942)
Supplement: Supplementary file 4 [file Table_4.docx]

**Supplement 4: Limited Data Extraction Form**

| **Query #** | **Query** | **Response Types** | **Notes & Inclusion/Exclusion Criteria** |
| --- | --- | --- | --- |
| 1 | Who is reviewing this article/document? | Open | [Note: Provide reviewer initials.] |
| 2 | What is the last name of the first listed author? | Open | [Note: Provide the last name only of the first listed author.] |
| 3 | What is the year of publication of the article/document? | Open | [Note: Provide the date of publication, **not** the date of acceptance. For articles with multiple publication dates (e.g., online, in print), use the earliest publication date.] |
| 4 | Was this a commentary, an empirical study, or some kind of literature review? | 0=commentary 1=empirical study 2=literature review |  |
| 5 | Where did the study take place? | If the article/document is empirical research or a review of empirical research, in what country(ies) does this research occur? | Open 0=NA |
| 6 | Provide the article purpose and/or project/study purpose or research question. | Open | [Note: Describe the purpose of the article. If the article primarily concerns a program or research study, provide the project/program's purpose or the research study's research question. If the article/study purpose is provided, quote it directly. Place direct quotes in quotation marks.] |
| 7 | List the health condition or issue that is addressed by the program or study described in the article. | Open 0=NA | [Note: Limit response to 1-2 words (e.g., "diabetes", "cancer"). Use "NA" if this is a program/study that does not target a specific health condition or set of health conditions.] |
| 8 | Does the article discuss governance of research? | 0=Yes 1=No |  |
| 9 | If you answered "yes" to question #8, briefly describe findings or discussion related to data governance and/or data stewardship. | Open 0=NA |  |
| 10 | Did the community participating in research employ its own methods for guiding/regulating research (e.g., research codes, tribal resolution, CAB)? | 0=Yes 1=No 2=NR | [Note: Use "NR" if it is unknown whether the community employed its own methods of research regulation.] |
| 11 | Does the article discuss data management or components of data management, including: data collection, storage, security, use, sharing, ownership, control, access, possession; databases/biobanks; data management tools; and, results dissemination. | 0=Yes 1=No | [Note: Data management tools include policies/protocols, groups, and systems/databases.] |
| 12 |  | Open 0=NA | [Note: Paraphrase or use direct quotes. Place direct quotes in quotation marks.] |
| 13 | Does the article describe the use or development of any data management tools? | 0=Y 1=N | [Note: Data management tools include policies/protocols, groups, and systems/databases.] |
| 14 | Does the article discuss community engagement in the research process or the use of a participatory research approach? | 0=Yes 1=No |  |
| 15 | If you answered "yes" to question #14, briefly describe findings or discussion related to community engagement. | Open 0=NA | [Note: Paraphrase or use direct quotes. Place direct quotes in quotation marks.] |
| 16 | Was (were) the research project(s) or program(s) described as employing a specific or a general participatory research approach? | 0=Specific participatory research approach 1=General participatory research approach 2=Non-participatory research approach 3=NR 4=NA | [Note: Use "specific" if the study or project/program is explicitly described as employing a specific participatory methodology (e.g., CBPR, TPR, PAR). Use "general" if the study or project/program is described as involving community engagement/participation/empowerment or a similar term, but does not explicitly describe use of a particular participatory research approach. Use "non-participatory" if a non-participatory approach is described. Use "NR" if the approach is unknown.] |
| 17 | Did the community participating in research employ its own methods for guiding/regulating research (e.g., research codes, tribal resolution, CAB)? | 0=Yes 1=No 2=NR | [Note: Use "NR" if it is unknown whether the community employed its own methods of research regulation.] |
| 18 | If you answered "Yes" to question #17, briefly describe the method. | Open 0=NA | [Note: Paraphrase or use direct quotes. Place direct quotes in quotation marks.] |
| 19 | Does the article present any original standards, guidelines, or recommendations related to governance, data management, or community engagement? | 0=Yes 1=No | [Note: SGR do not include documents to regulate research (e.g., research codes, MOA/MOUs, MDSAs). SGR refer to the broader rules and principles that should guide research. They are not specific to a given community or research project.] |
| 20 | If you answered "Yes" to question #19, briefly describe the standards, guidelines, or recommendations. | Open 0=NA | [Note: Paraphrase or use direct quotes. Place direct quotes in quotation marks.] |
| 21 | Provide any information relevant to the research question that is not captured by the preceding queries. | Open | [Note: Paraphrase or use direct quotes. Place direct quotes in quotation marks.] |
